# Rational Design and Fabrication of MEMS Gas Sensors With Long‐Term Stability: A Comprehensive Review

**DOI:** 10.1002/advs.202511555

**Published:** 2025-09-12

**Authors:** Chao Zhang, Tianyi Wang, Guozhu Zhang, Rui Gao, Chengze Gao, Zeyu Wang, Fuzhen Xuan

**Affiliations:** ^1^ Shanghai Key Laboratory of Intelligent Sensing and Detection Technology School of Mechanical and Power Engineering East China University of Science and Technology Shanghai 200237 P. R. China

**Keywords:** device structure optimization, long‐term stability, machine olfactory, MEMS gas sensor, sensing film degradation

## Abstract

With the growing demand for chemical information collection from the environment, miniaturized size and high sensitivity labeled microelectromechanical systems (MEMS) gas molecular sensing devices have emerged as a promising element in the development of machine olfactory. However, prolonged exposure to gas analytes often induces slow chemical transformations on the sensing film surface, leading to reduced chemical activity, performance degradation, and mechanical failures such as membrane cracking or delamination. In the real market, especially under harsh working environments, long‐term stability is a critical quality metric in gas sensor development. Therefore, the pursuit of MEMS gas sensors that offer both high sensitivity and extended lifespan becomes indispensable yet challenging. Thus, this review provides a comprehensive overview of recent studies and achievements in MEMS gas sensors, highlighting efforts aimed at enhancing their working stability. Key areas of focus include advancements in the chemical and physical characteristics of sensing films, as well as improvements in device structures. Furthermore, current limitations, perspectives, and future possibilities for designing and fabricating MEMS gas sensors with long‐term stability are discussed.

## Graphical abstract

Recent advances in microelectromechanical systems (MEMS) gas sensors are reviewed, emphasizing strategies to enhance long‐term stability, including chemical modification of sensing films and device structural optimization. Key challenges and future opportunities are also highlighted.

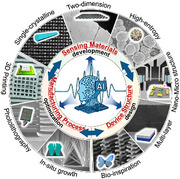

## 1 Introduction

The incorporation of microelectromechanical systems (MEMS) technology into gas molecular sensor design and fabrication has led to transformative changes to the commercialization of gas sensors over the past decade.^[^ ^1^, ^2^, ^3^, ^4^ ^]^ According to IDTechEx, a renowned market research firm, the market size for gas sensors is projected to reach 9.5 billion US dollars by 2034.^[^ ^5^ ^]^ This substantial market demand highlights the significant application prospects and economic value of developing high‐performance MEMS gas sensors and associated molecule recognition electronics. Compared to conventional tube‐type or print‐type gas sensors, MEMS‐based sensing devices offer numerous advantages, such as compact size, low power consumption, cost‐effectiveness, and high sensitivity.^[^ ^6^, ^7^, ^8^, ^9^ ^]^ These attributes make them ideal candidates for achieving the widespread digitization of the environment. To accomplish this goal, the key aspect of MEMS gas sensor development lies in the precise and real‐time transduction of chemical information from gas species. As a result, the fabricated sensors must exhibit high sensitivity and long‐term stability. Although sensitivity has received extensive attention during the development of MEMS gas sensors,^[^ ^10^, ^11^, ^12^ ^]^ the issue of long‐term stability has not been discussed as frequently. However, it remains a critical performance parameter for gas sensors to enter the market successfully. Leading sensor manufacturers like Bosch and Honeywell demand that the gas sensors they produce and manufacture must operate stably for more than 43 800 h (equivalent to 5 years).^[^ ^13^ ^]^ Furthermore, the U.S. Department of Energy's 2015 standards for hydrogen safety utilization require sensors to have a working lifespan of up to 10 years while ensuring rapid and accurate detection of H_2_.^[^ ^14^ ^]^ Consequently, the development of MEMS gas sensors with extended lifespans has emerged as a prominent topic in the commercialization process, given the significant demand and stringent requirements in various applications.

As known, MEMS gas sensors operate based on the principle that gas‐solid reactions induce changes in the electrical,^[^ ^15^, ^16^, ^17^ ^]^ optical,^[^ ^18^, ^19^ ^]^ or mechanical properties^[^ ^20^, ^21^, ^22^, ^23^ ^]^ of the sensing film. However, over extended periods of operation, the reactivity of the sensing film weakens due to the presence of target gas molecules with inherent reducibility or oxidizability, as well as interference gas molecules (e.g., H_2_O, O_2_, and CO_2_) in the background.^[^ ^24^, ^25^, ^26^ ^]^ This weakening leads to a transformation in the chemical structure of the sensing film, resulting in a degradation of the sensing performance of sensors. Moreover, to achieve high sensitivity in molecule detection, most MEMS gas sensors are integrated with a micro hot plate that provides temperature excitation.^[^ ^27^, ^28^, ^29^, ^30^ ^]^ However, prolonged exposure to high‐temperature heating can cause unexpected crystal growth of the sensing materials. The accompanying lattice expansion and contraction lead to stress concentration at the interface between the sensing film and the substrate, eventually causing cracks to initiate and diffuse.^[^ ^31^ ^]^ Consequently, the sensing film may detach from the substrate, leading to the failure of the sensor device. Thus, obtaining high sensitivity in gas molecule detection often comes at the cost of sacrificing the device's long‐term stability.

To address this challenge, significant efforts have been devoted to achieving both high sensitivity and stability in MEMS gas sensors, and the primary option is to enhance the chemical stability of the sensing materials.^[^ ^32^ ^]^ For example, metal oxides show promise for the electrical detection of gas species due to their environmentally tolerant chemical stability.^[^ ^33^, ^34^, ^35^ ^]^ However, despite their excellent chemical stability, metal oxides can still be vulnerable to damage from water, CO_2_, and other environmental pollutant gases after prolonged exposure, resulting in surface degradation.^[^ ^36^, ^37^ ^]^ Consequently, several approaches, such as thermal treatment,^[^ ^38^ ^]^ doping,^[^ ^39^ ^]^ and the addition of filter membranes,^[^ ^40^ ^]^ are proposed to enhance their chemical stability. Additionally, electroactive polymers serve as excellent sensing elements for the integration of MEMS gas sensors.^[^ ^41^ ^]^ However, over an extended period of exposure to the environment, the functional groups and carbon chains in these polymers are prone to oxidation.^[^ ^42^ ^]^ To mitigate this degradation, a recent approach involves the introduction of antioxidizing agents.^[^ ^43^ ^]^ In addition to chemical stability, enhancing the thermal stability of the sensing film is crucial for the reliable operation of gas sensors. Metal oxide sensing elements typically require elevated temperatures to activate gas sensing. However, prolonged exposure to elevated temperatures during gas sensing can lead to crystal growth and conductivity drift in the sensing film, impacting performance. To address this, single‐crystal integrated MEMS sensor devices, like nanowires or nanosheets fabricated using photolithography, replace conventional polycrystalline sensor membranes, resulting in improved thermal stability.^[^ ^44^ ^]^ This approach advances high sensitivity and long‐term stability in MEMS gas sensors, providing more reliable gas sensing performance even under demanding thermal conditions. Besides material stability, integrating sensor devices with optimized mechanical structures is considered another important approach to improve the lifespans of MEMS gas sensors. For instance, palladium (Pd) is widely used in designing room‐temperature H_2_ sensors due to its exceptional hydrogen storage characteristics and thermal/chemical stability.^[^ ^45^ ^]^ However, multiple cycles of H_2_ adsorption/desorption can cause Pd lattice expansion and contraction, leading to lattice distortion known as “hydrogen embrittlement.”^[^ ^46^ ^]^ This compromises the mechanical stability of the Pd sensing film, causing it to delaminate from the substrate and result in device failure. To address this issue, several studies have demonstrated that multilayer Pd‐based electrode structures can effectively suppress hydrogen‐induced stress accumulation.^[^ ^47^ ^]^ These configurations serve as strain‐buffering architectures, accommodating the cyclic lattice expansion and contraction that occurs during hydrogen adsorption and desorption. By dispersing and relieving localized mechanical stress, they significantly enhance the long‐term operational stability of the devices.

As is evident, despite significant efforts having been made to enhance the stability of MEMS gas sensors, numerous challenges persist when these sensors are employed in harsh environments, such as high temperatures, high pollution levels, and the extreme conditions of radiative space. In this review, we offer a comprehensive state‐of‐the‐art analysis of MEMS gas sensor design and fabrication, with a specific focus on achieving long‐term stability. Specifically, we provide an overview of recent MEMS gas sensors, as well as their corresponding sensing mechanisms. After a brief summary of MEMS gas sensors, we will present a detailed discussion of recent progress on the design and fabrication of typical MEMS gas sensors. Then, we emphasize the discussion of the origins of the instability in MEMS gas sensors and the approaches applied to improve sensor stability. Finally, we summarize recent progress that has aimed to enhance sensor stability and provide an outlook on the design and fabrication of MEMS gas sensors, focusing on achieving both high sensitivity and a long lifespan. This review is expected to inspire the design of high‐performance MEMS gas sensors with long‐term stability to meet the demands in harsh environments.

## 2 Evolution of MEMS Gas Sensors

**Figure 1:**
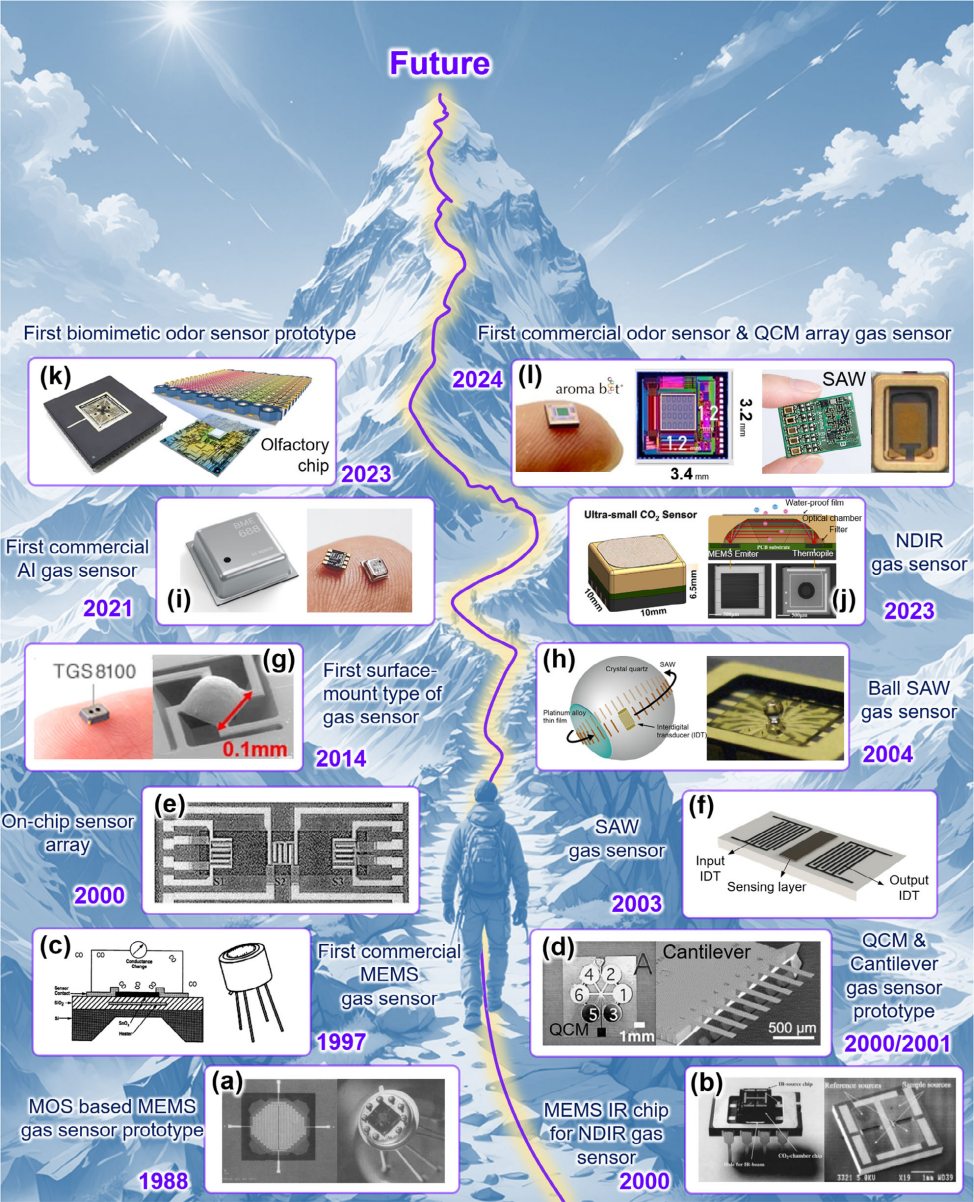
Roadmap of MEMS gas sensors.

The modern gas detection technology has deep and significant roots, with a pivotal milestone dating back to 1926.^[^ ^48^ ^]^ During this time, Dr. Oliver Johnson innovatively developed the catalytic combustion sensor, a revolutionary breakthrough that fundamentally transformed the field of gas detection. This development marked the transition from traditional empirical methods to advanced electronic detection. Subsequently, in 1962, Seiyama and colleagues introduced conductometric gas detection technology based on ZnO semiconductor films, injecting new vitality into gas detection research.^[^ ^49^ ^]^ This innovation spurred the development of various novel gas sensors, encompassing optical, acoustic, and mechanical types, thereby greatly enriching the methodologies for gas detection and identification. Meanwhile, in the 1960s, the advent of silicon micromachining technology acted as a powerful catalyst, significantly accelerating the rapid development of microelectromechanical systems (MEMS), especially in sensor electronics, where its impact was particularly profound.^[^ ^50^ ^]^ During this period, Demarne and Grisel first employed advanced CMOS processes to successfully integrate a microheater into metal oxide conductometric gas sensors on diminutive silicon chips via an etching method,^[^ ^51^ ^]^ as shown in **Figure** **1**a. This groundbreaking achievement not only highlighted the immense potential of MEMS technology but also marked the official inception of the MEMS gas sensor prototype. This milestone established a solid foundation for the subsequent miniaturization, integration, and intelligent advancement of gas detection technology.

After more than a decade of technological refinement and iteration, Motorola set the trend in 1997 by introducing the TO‐4 packaged metal oxide conductometric CO gas sensor, manufactured using silicon micromachining techniques,^[^ ^52^ ^]^ as shown in Figure 1c. This product not only marked the commercialization of MEMS gas sensors but also signaled a significant advancement in gas detection technology. Concurrently, the rapid development of silicon micromachining technology provided robust support for the prototype design of MEMS light emitters. Bauer and colleagues successfully proposed such prototypes in 1996, significantly advancing the miniaturization of NDIR (nondispersive infrared) optical gas sensors (Figure 1b).^[^ ^53^ ^]^ Moreover, innovative concepts based on mechanical signal changes induced by gas molecule adsorption effects led to the creation of various new MEMS gas sensor prototypes, including quartz crystal microbalances (QCM),^[^ ^54^ ^]^ cantilevers,^[^ ^55^ ^]^ and surface acoustic waves (SAW)^[^ ^56^ ^]^ (Figure 1d,f). These developments collectively enriched the technical framework of MEMS gas sensors. However, practical applications often face significant interference from numerous gas components, rendering single sensors inadequate in achieving high selectivity for target gas molecules. To address this challenge, Cané et al.^[^ ^57^ ^]^ introduced a novel MEMS sensor array structure integrating multiple sensor units, as shown in Figure 1e. This innovative design incorporates units with diverse sensitive characteristics, enabling a more comprehensive and precise capture of complex gas compositions. Moreover, by employing advanced machine learning algorithms, the system intelligently analyzes data from each sensor unit, thereby facilitating accurate detection and identification of target gases in complex environments. This advancement promises to significantly broaden the application prospects of gas detection technology. In advancing MEMS gas sensors towards widespread deployment, alongside selectivity, structural size, and power consumption have emerged as critical considerations. Addressing these challenges, Figaro Corporation pioneered the integration of surface‐mount packaging technology into MEMS gas sensors in 2014^[^ ^58^ ^]^ (Figure 1g). This initiative effectively reduced sensor dimensions to the millimeter scale, facilitating high integration and portability. Moreover, it significantly decreased power consumption to the milliwatt level, thereby greatly extending device lifespan. This innovation represents a significant milestone in the evolution of MEMS gas sensor technology. Simultaneously, the diversification of MEMS sensor technology has exhibited robust growth. In 2004, Yamanaka et al.^[^ ^59^ ^]^ introduced an innovative spherical MEMS SAW gas sensor, as shown in Figure 1h. This unique design enables efficient propagation of gas response signals along the equatorial direction of the sphere. When equipped with a palladium‐based sensitive membrane, this sensor achieves precise detection of hydrogen within the concentration range of 10 ppm to 100%, showcasing exceptional sensitivity and detection capability. In 2023, Li et al.^[^ ^60^ ^]^ first proposed an integrated design method for NDIR gas sensors, as shown in Figure 1j. The fabricated sensor device features a high‐emissivity MEMS emitter chip, a Si optical cell with high coupling efficiency, and a thermopile chip with high detectivity, all integrated into a compact 10 mm × 10 mm × 6.5 mm package. This represents an approximately 80% reduction in size compared to typical commercial sensors, offering advantages such as low power consumption and fast response speed.

In the past decade, propelled by rapid advancements in artificial intelligence (AI) technology, the field of gas sensors has encountered transformative opportunities. Researchers have embarked on exploring methods to achieve real‐time and precise output of gas types and concentrations during gas detection to meet increasingly complex application requirements. Against this backdrop, Bosch introduced the BME‐688 MEMS gas sensor in 2023, incorporating AI computing capabilities^[^ ^61^ ^]^ (Figure 1i). This sensor not only inherits the advantages of MEMS technology, such as miniaturization and low power consumption, but also employs embedded AI algorithms for intelligent processing and analysis of gas molecule information. It is capable of promptly providing detailed information on gas types and concentrations, thereby opening new avenues for intelligent and automated applications in gas detection technology. The BME688 is the first gas sensor with Artificial Intelligence (AI), which is housed in a robust yet compact 3.0 × 3.0 × 0.9 mm^3^ package and especially developed for mobile and connected applications where size and low power consumption are critical requirements. The gas sensor can detect Volatile Organic Compounds (VOCs), volatile sulfur compounds (VSCs), and other gases such as carbon monoxide and hydrogen in the parts‐per‐billion (ppb) range. In the continuous pursuit of optimal gas sensor technology, emulating biological olfactory systems has emerged as a common aspiration among researchers. To develop odor sensors with high sensitivity and selectivity, integrated with storage and computing functions for real‐time capture and feedback of environmental chemical information, Fan's research team achieved a significant breakthrough in 2023.^[^ ^62^ ^]^ They successfully engineered an advanced biomimetic olfactory chip that cleverly integrates an impressive array of 10 000 micro gas sensors within a compact 8 mm^2^ footprint, as shown in Figure 1k. This chip exhibits outstanding performance, enabling precise detection and identification of mixed gases while demonstrating exceptional discrimination capability across up to 24 distinct odors. This achievement marks a significant stride towards bridging gas sensing technology with biological olfactory systems. Continuing along this path of innovation, Aroma Bit, a Japanese company, achieved notable advancements in the following year, 2024.^[^ ^63^ ^]^ They successfully developed odor imaging sensors based on CMOS semiconductor technology and prototypes of SAW array olfactory sensors, as shown in Figure 1l. Particularly noteworthy is the odor imaging sensor, featuring a sensing area of just 1.2 mm × 1.2 mm densely populated with 57 600 sensing elements. This remarkable density not only establishes it as the world's smallest and most densely packed electronic nose‐type olfactory sensor but also underscores the substantial potential of gas detection technology in the realms of miniaturization and high precision. In addition, Aroma Bit's introduction of the SAW array olfactory sensor is equally noteworthy. This sensor integrates five MEMS SAW sensing units, each compact in size (1.2 mm × 1.2 mm) yet demonstrating powerful detection capabilities. It achieves precise identification and differentiation of nearly 20 different gases. This accomplishment not only highlights the distinctive advantages of SAW technology in gas sensing but also provides robust support for future applications requiring high‐precision, multi‐gas detection.

In the future, with the continuous advancements in micro/nano fabrication techniques and the rapid development of sensor signal processing capabilities, MEMS gas sensors will evolve towards miniaturization, low power consumption, integration of multiple sensing elements on a single chip, and the integration of sensing, computing, and storage. These advancements will pave the way for intelligent olfactory chips, enabling real‐time detection and rapid recognition of odor molecules in the environment.

## 3 Typical MEMS Gas Sensors

From the development trajectory of MEMS gas sensors, they can primarily be classified into electrochemical, optical, and mechanical types based on their molecular sensing principles. Among these, electrochemical MEMS gas sensors have garnered significant attention due to their simple structure, ease of miniaturization, and high sensitivity, marking them as a notable success in commercial applications over the past decades.^[^ ^64^, ^65^, ^66^ ^]^ Additionally, NDIR‐type optical gas sensors and SAW/QCM‐type mechanical MEMS gas sensors are also considered promising for specific gas molecule detection, including CO_2_ and analysis of explosive or toxic chemicals.^[^ ^67^ ^]^ In this section, we will delve into the discussion of the sensing principles and their integration structure of these typical MEMS gas sensors.

### 3.1 Electrochemical Type of MEMS Gas Sensor

The working principle of electrochemical sensors is based on the chemical interactions between target molecules and the surface of the sensing film, leading to variations in electrical conductivity.^[^ ^68^ ^]^ Specifically, these fluctuations in electrical signals are recorded and analyzed to identify the type of exposed gas and quantify its concentration. Therefore, the sensing film is considered a critical component of electrochemical MEMS gas sensors. The sensing mechanisms of electrochemical gas sensors have been extensively studied in recent research.^[^ ^69^ ^]^ These sensors typically consist of a two‐ or three‐electrode system and an ionic conductor, detecting gas concentrations through oxidation or reduction reactions occurring at the working electrode. During this process, ions migrate through the electrolyte while electrons flow through an external circuit, generating a measurable current. This mechanism enables electrochemical sensors to exhibit high sensitivity in detecting toxic gases such as carbon monoxide (CO) while maintaining low power consumption and excellent portability.^[^ ^70^ ^]^

**Figure 2:**
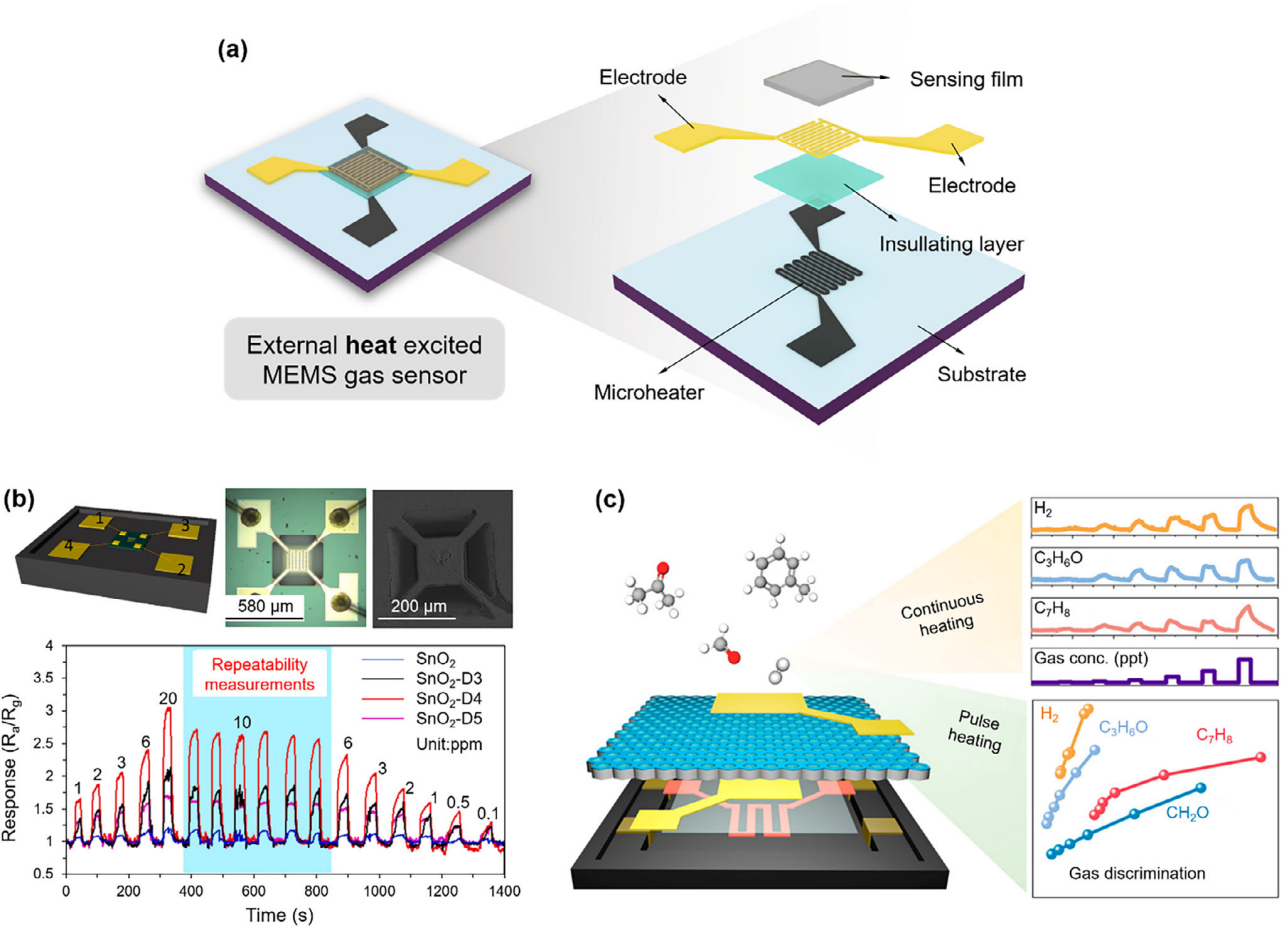
MEMS gas sensors with external heat‐excited sensing structures. a) Typical structure. b) Fabrication of SnO_2_ with oxygen vacancy defects (SnO_2_‐D), and dynamic response‐recovery curves of the MEMS sensor to different concentrations of H_2_ at 250 °C. Reproduced with permission.^[^ ^73^ ^]^ Copyright 2022, Elsevier. c) The dual‐mode microheater‐integrated nanotube array (MINA) gas sensor capable of ultra‐low detection limits (down to ≈7 ppt) and selective gas discrimination using transient feature extraction in pulse heating mode. Reproduced with permission.^[^ ^74^ ^]^ Copyright 2022, American Chemical Society.

**Figure 3:**
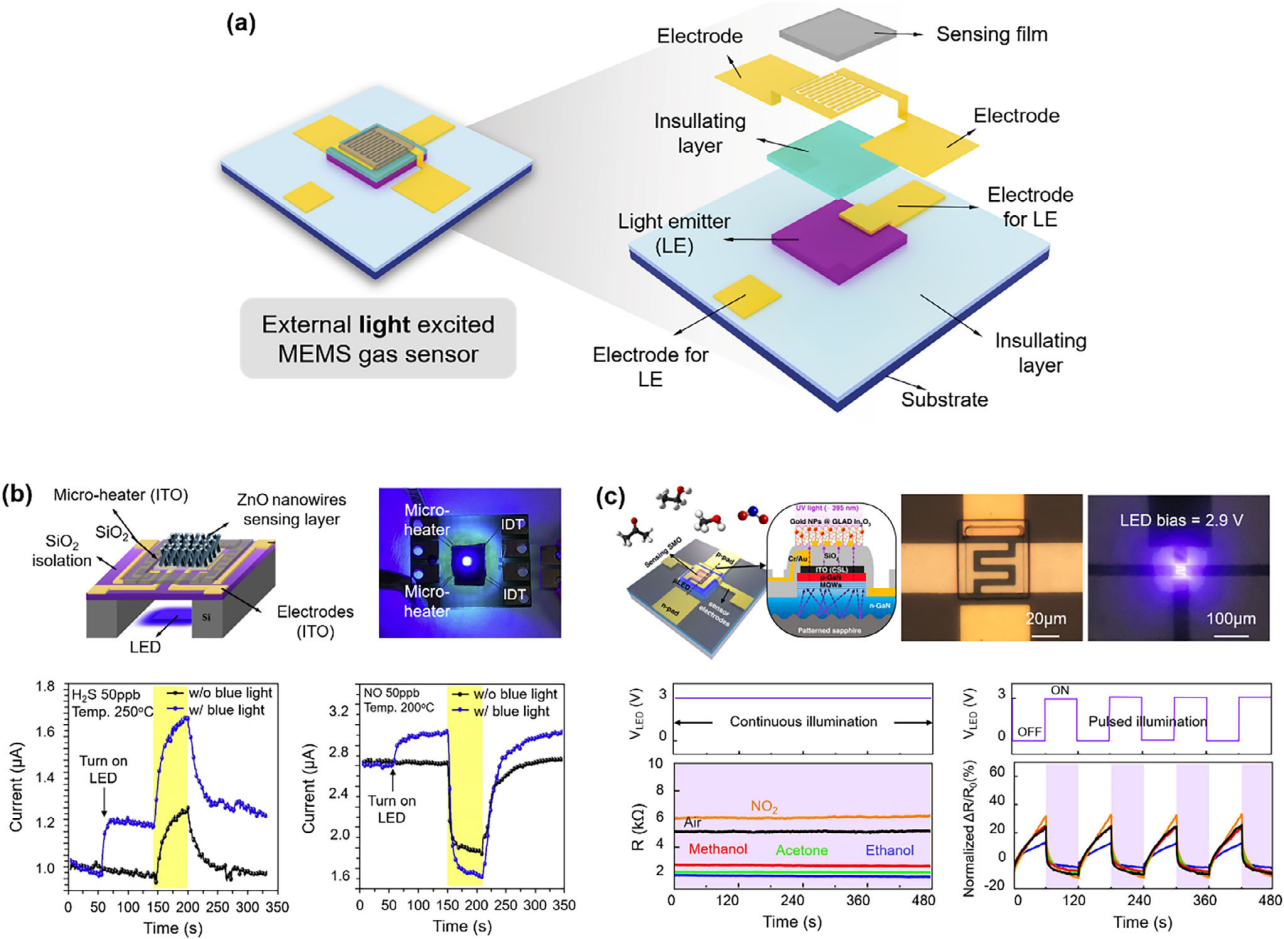
MEMS gas sensors with external light‐excited sensing structures. a) Typical structure. b) The structure of the ZnO nanowire MEMS gas sensor and transient current response to H_2_S and NO with and without blue light illumination. Reproduced with permission.^[^ ^75^ ^]^ Copyright 2020, Elsevier. c) The structure of the µLED‐embedded photoactivated (µLP) gas sensor and its calibration curves, as well as dynamic responses to various gases under continuous and on–off µLED illumination. Reproduced with permission.^[^ ^78^ ^]^ Copyright 2023, Springer Nature.

Functional nanomaterials, including metals, semiconductors, and conducting polymers, have been widely explored as key sensing materials for electrochemical gas sensors due to their excellent electrochemical properties. To further enhance the performance of electrochemical MEMS gas sensors, external excitation mechanisms, such as microheaters or micro light sources, are often incorporated into their design and fabrication (**Figures** **2**a and **3a**). This necessity arises from the strong correlation between the reactivity of gas molecules on sensing materials and the surface activity of these materials, which can be effectively modulated by temperature or light exposure. High temperatures or high‐energy photons reduce the activation energy barrier for chemical reactions between gas molecules and the sensing film, thereby accelerating electron exchange processes, enhancing gas adsorption and desorption rates, and enabling the sensor to respond to target gases more rapidly and reliably. For instance, Bindiya Dey et al.^[^ ^71^ ^]^ synthesized cobalt‐substituted ferrite nickel nanoparticles using a hydrothermal method and demonstrated that certain materials require specific temperature conditions to achieve optimal sensing performance. Similarly, Geng's research on the electrochemical gelation of metal chalcogenide quantum dots revealed that gel‐based sensitive materials exhibit outstanding performance in high‐surface‐area applications such as photocatalysis and gas sensing. These findings further support the necessity of external thermal or light excitation in improving the performance of electrochemical gas sensors.^[^ ^72^ ^]^ The microheater is usually made of a material with high electrical resistance, primarily platinum (Pt), and is patterned into a serpentine shape using lithography techniques on a silicon substrate. To minimize heat loss and enhance efficiency, the microheater is typically placed in close proximity to the sensing film. A typical structure of an electrochemical MEMS gas sensor is shown in Figure 2a. A Pt microheater is first deposited on the silicon substrate, and then a sensing film is deposited above the microheater after inserting an insulating layer. Based on this structure, versatile MEMS gas sensors composed of functional nanomaterials are proposed. As an example, Luo et al.^[^ ^73^ ^]^ developed a low‐power SnO_2_ MEMS gas sensor, which consumed only 25 mW at a working temperature of 200 °C (Figure 2b). Additionally, Tang et al.^[^ ^74^ ^]^ developed a dual‐mode microheater‐integrated nanotube array (MINA) gas sensor (Figure 2c). This sensor features a high‐efficiency microheater that supports two operational modes: continuous heating (CH) for stable and highly sensitive gas detection at ppt levels, and pulse heating (PH) for extracting transient response features, enabling selective gas discrimination without the need for sensor arrays.

In addition to using a microheater to generate high temperatures for enhancing gas molecular sensitivity, light irradiation represents another efficient method to achieve superior sensing performance. This type of electrochemical gas sensor integrates a light emitter instead of a microheater in the device structure, as depicted in Figure 3b.^[^ ^75^ ^]^ More importantly, photoactivation enables the detection of gas molecules at room temperature while preventing thermal damage to the gas sensor platform. Previously, external light sources such as xenon lamps or pre‐packaged light‐emitting diodes (LEDs) positioned above or below the sensor device were commonly used for integrating photoactivated gas sensors.^[^ ^76^ ^]^ However, this approach often results in inefficient photon energy transfer, leading to high power consumption and difficulties in miniaturization. In 2019, Markiewicz et al.^[^ ^77^ ^]^ proposed a photoactivated NO_2_ gas sensor directly mounted onto a blue LED, introducing a new strategy for on‐chip integration of sensing elements and light sources. In 2020, Cho et al.^[^ ^78^ ^]^ introduced the first monolithically integrated photoactivated gas sensors combined with µLED platforms (µLPs), as shown in Figure 3c. Within this sensor device, GaN‐based µLPs were successfully fabricated using standard industrial‐grade microfabrication processes. Gas‐sensitive ZnO nanowires were then synthesized directly on top of the fabricated µLPs through hydrothermal synthesis. Such a sensor design offers not only a more miniaturized device size but also a low power consumption (≈184 µW) and excellent gas sensitivity (Δ*R*/*R*_0_ = 605% to 1 ppm NO_2_). This significant development introduces a promising new option for advancing photoactivated MEMS gas sensors, highlighting its potential for practical applications in mobile Internet of Things (IoT) devices.

To achieve high‐performance electrochemical MEMS gas sensors, the choice of sensing material is also crucial. Among the nanomaterials used as sensing layers for electrochemical gas sensors, metal oxide semiconductors (MOS) have garnered intensive research attention due to their high molecular sensitivity, rapid response and recovery rates, and robust chemical and thermal stability. Traditional MEMS gas sensors typically employ chemical/physical thin‐film deposition techniques (such as sputtering, chemical vapor deposition, and sol–gel methods) to form thin‐film sensing layers, ensuring high reproducibility and mechanical stability.^[^ ^79^ ^]^ In recent years, with the continuous advancement of nanomaterial synthesis technology and micro/nano fabrication processes, researchers have developed single nanosheets or independent micro/nano structures that can be directly grown in situ on the surface of MEMS devices, thereby constructing sensitive layers for novel electrochemical gas sensors with excellent performance.^[^ ^80^, ^81^ ^]^ Common in situ growth methods include hydrothermal/solvothermal synthesis, chemical vapor deposition (CVD), and electrochemical deposition.^[^ ^82^ ^]^ Among these, the hydrothermal method offers advantages such as mild reaction conditions and simple equipment, making it suitable for synthesizing highly crystalline nanostructures at relatively low temperatures. It is widely used for constructing one‐dimensional or two‐dimensional nanostructures of metal oxides like ZnO and SnO_2_. The CVD method, on the other hand, is more suitable for preparing high‐purity, structurally controllable nanofilms, particularly demonstrating significant advantages in the synthesis of two‐dimensional transition metal sulfides such as MoS_2_ and WS_2_.

Compared to traditional spin‐coating or sol–gel processes, in situ growth offers notable advantages in long‐term stability. This is primarily because it eliminates the need for pre‐dispersing nanomaterials, avoids particle agglomeration and solvent residues, and enables direct film formation while preserving the original nanostructures.^[^ ^83^, ^84^, ^85^ ^]^ The resulting sensitive layers typically possess higher specific surface areas and active site densities, along with denser and more uniform film morphologies. These features enhance gas adsorption efficiency and charge transport, thereby improving the device's response intensity, sensitivity, and selectivity.^[^ ^86^ ^]^ Additionally, the in situ growth of sensitive materials on the surface of electrodes or MEMS substrates can form stronger interfacial bonds, significantly reducing signal loss caused by interfacial resistance and poor contact. This provides a continuous and stable conduction path for charge transport, contributing to improved stability and reproducibility of the device during prolonged operation.^[^ ^87^, ^88^, ^89^ ^]^

Despite the many advantages of in situ growth technology, its practical application still faces several challenges. First, some processes (such as CVD) require high processing temperatures (typically exceeding 500 °C), which may cause thermal damage to temperature‐sensitive MEMS substrate materials (such as polyimide or low‐temperature glass), limiting their material compatibility. Second, the in situ growth process is highly sensitive to process parameters, especially during vapor deposition, where the temperature field, precursor distribution, and gas flow stability can all affect the thickness uniformity of the film. Achieving high consistency over large‐area deposition remains a challenge. More importantly, due to the mismatch in thermal expansion coefficients between the deposited film and the substrate, residual stress can be introduced during high‐temperature deposition, leading to film cracking or delamination, which severely impacts the long‐term reliability of the device.^[^ ^90^, ^91^ ^]^ Furthermore, compared with traditional batch processes, in situ growth technology still lacks sufficient automation and scalability, hindering its industrial‐scale adoption. Therefore, future research efforts should focus on enhancing low‐temperature compatibility, controlling film uniformity, developing stress regulation strategies, and improving process scalability to promote the widespread application of in situ growth methods in high‐performance MEMS gas sensors.

### 3.2 Optical MEMS Gas Sensor

**Figure 4:**
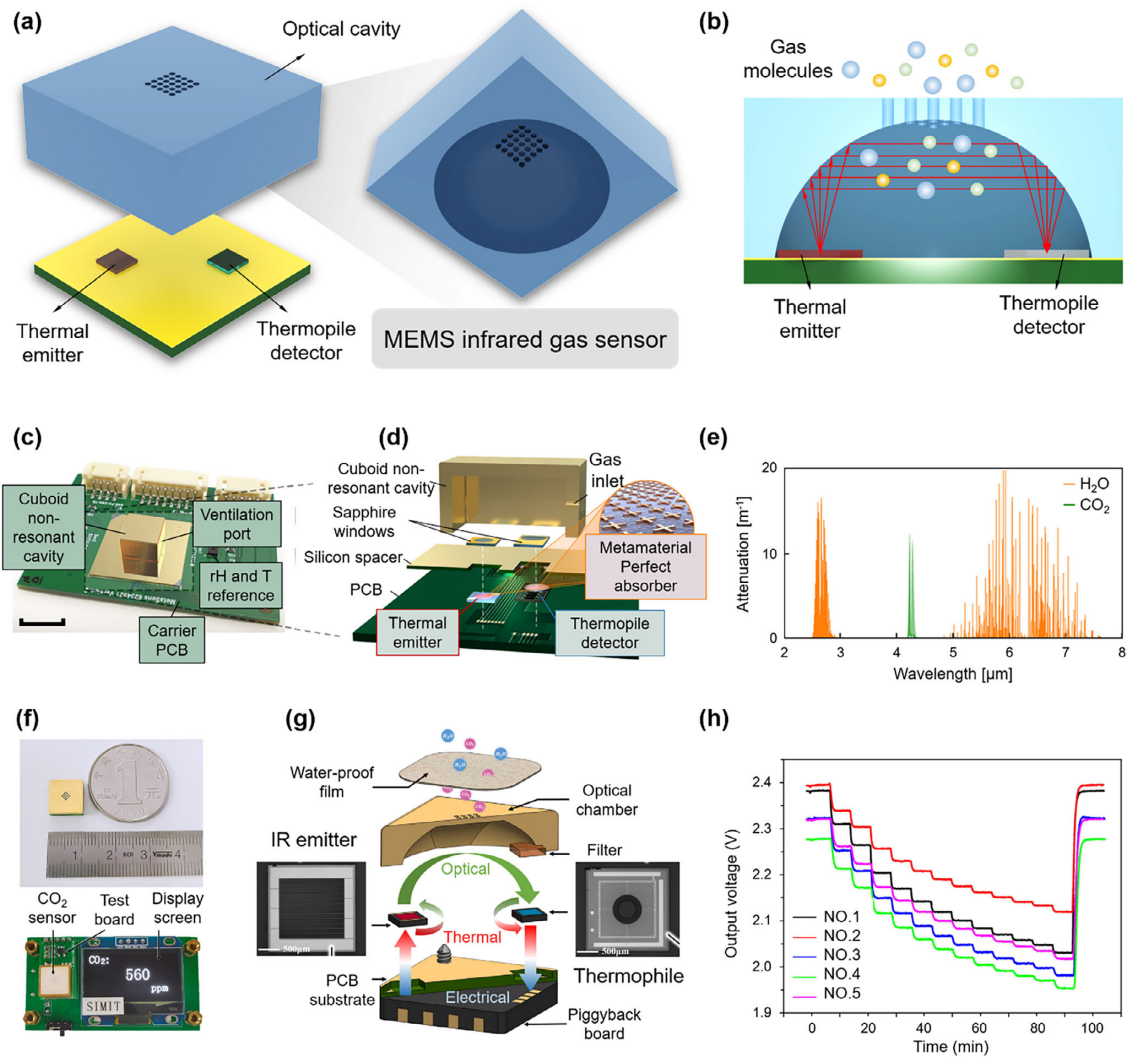
Applications of optical gas sensors. a) Typical NDIR sensor structure. b) The cross‐sectional structure of the CO_2_ sensor and its infrared absorption principle. c–e) The structure of the miniaturized mid‐infrared CO_2_ sensor using plasmonic metamaterials as on‐chip optical filters, along with the attenuation spectra of CO_2_ and H_2_O under ambient conditions. Reproduced with permission.^[^ ^92^ ^]^ Copyright 2020, American Chemical Society. f–h) The overall design of the CO_2_ sensor, the energy conversion process, and the output voltage of the five sensors. Reproduced with permission.^[^ ^60^ ^]^ Copyright 2023, Elsevier.

An optical gas sensor is a type of sensing platform that detects changes in the optical properties of light as it interacts with gas molecules. In contrast to conventional free‐space optical sensor systems and fiber sensors, MEMS optical gas sensors usually employ meticulously designed nano/microstructures to enhance light‐molecule interactions. Although these sensors can achieve highly sensitive detection of gas molecules, they still pose significant challenges for device design. These challenges primarily include the creation of spectrally efficient light sources, devices integrated with waveguides to minimize optical losses, and photodetectors that offer high sensitivity. Among recent diverse optical gas detection platforms discussed, NDIR gas sensors stand out as successful candidates for on‐chip integration due to their straightforward construction and remarkable sensitivity. An NDIR optical gas sensor typically comprises a light source that can emit IR light, a gas cell that provides a controlled environment for the interaction between IR light and gas molecules and a detector which is sensitive to the emitted IR light, as shown in **Figure** **4**a and 4b. The operational principle of NDIR sensors is grounded in Beer‐Lambert's law, which stipulates a linear relationship between the intensity of light absorbed by gases and the concentration of the target gas. Through precise measurement of subtle variations in absorbed light intensity, NDIR sensors provide accurate concentration information for target gases. Therefore, current advancements in NDIR‐type MEMS gas sensors focus on designing and fabricating high‐efficiency IR emitters, optimizing the gas cell for high coupling efficiency to ensure minimal infrared light loss, and developing IR detectors with high sensitivity.

MEMS microheaters have recently gained attention for NDIR gas sensors due to their high energy efficiency, greater reliability, faster response, low thermal mass, smaller size, and compatibility with the CMOS foundry process. Leuthold et al.^[^ ^92^ ^]^ designed a CO_2_ gas sensor (0–500 00 ppm CO_2_) which utilizes a thermal mid‐IR source by combining a MEMS heater with metamaterial perfect absorber structures (Figure 4c–e). The structure of the emitter has a great impact on the performance since the device showed a 5‐fold enhancement (at a fixed emitter‐to‐detector distance of 4 mm and a thermopile detector) in relative sensitivity as compared to the blackbody emitter‐based sensor. Regarding the gas cell in an NDIR gas sensor, its primary objectives are to provide a confined path for emitted infrared radiation to reach the detector without being influenced by external environmental factors, to create a controlled environment for interaction with the target gas, and to support the overall gas sensing system structure. A cavity optical cell is a structure that enables light reflection in a confined space, which can be easily fabricated using CMOS techniques compared to conventional face‐to‐face and planar cells. Feng et al.^[^ ^60^ ^]^ developed an ultra‐small NDIR CO_2_ sensor integrating a MEMS emitter, a thermopile detector, and an optical chamber. The sensor is only 10 mm × 10 mm × 6.5 mm in size (Figure 4f–h), making it 80% smaller than commercial models, with advantages of low power consumption and fast response speed. The role of the IR detector in NDIR gas sensors is to convert electromagnetic radiation energy or temperature differences, after interaction with gas molecules, into readable electrical signals. Recently, a number of IR detectors have been reported for NDIR gas sensors, which can be classified as thermopile detectors, pyroelectric detectors, photodiodes, and bolometers.

In addition to NDIR‐type MEMS gas sensors, significant efforts have been made to develop various MEMS‐based optical sensors, including those based on Raman spectroscopy, cavity ring‐down spectroscopy, and photothermal spectroscopy. However, current research on these optical MEMS sensors primarily focuses on the design and construction of optical cells or waveguides, rather than achieving full sensor integration in a compact form. Although high light–molecule interaction can be realized through carefully designed optical cells, these systems still face challenges such as large size, high cost, and difficulties with integration, largely due to the specific requirements for light sources and detectors.

### 3.3 Mechanical MEMS Gas Sensor

Mechanical gas sensors are a class of sophisticated sensing devices that operate based on capturing the mechanical signal changes induced by the adsorption of gas molecules onto sensitive elements. Specifically, when specific gas molecules attach to the surface of the sensitive layers, their presence significantly alters the mechanical properties of the unit, including aspects such as mechanical response, deformation extent, and vibration frequency. Subsequently, through precise conversion mechanisms, these subtle mechanical changes are accurately transformed into readable electrical signals, enabling effective detection and recognition of gas molecules.

**Figure 5:**
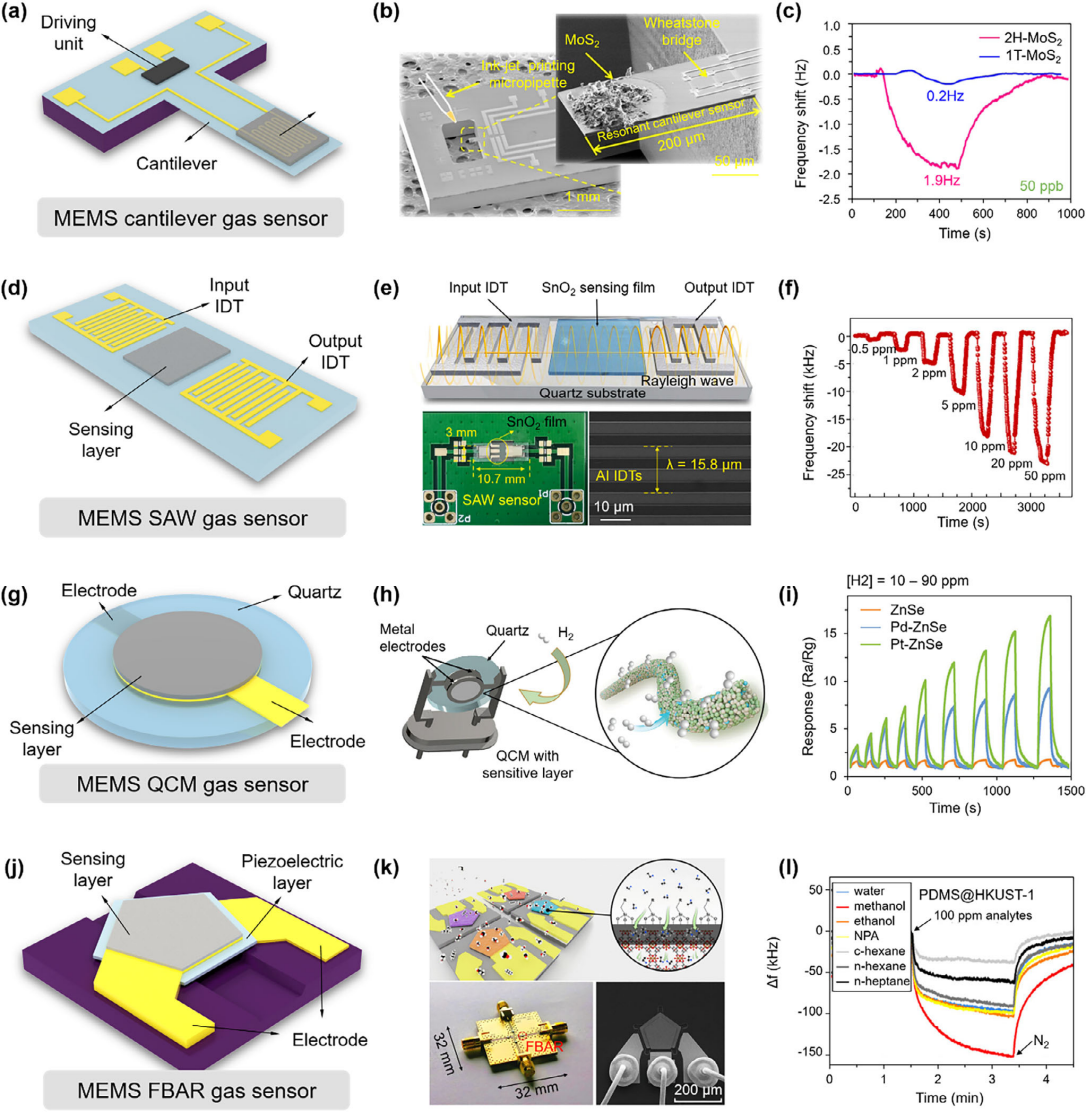
Structures and applications of mechanical MEMS gas sensors. a) The structure of the MEMS cantilever gas sensor. b,c) Fabrication of two‐dimensional MoS_2_ nanosheet‐based microcantilever sensors and their long‐term stability in resonance frequency response curves. Reproduced with permission.^[^ ^94^ ^]^ Copyright 2022, American Chemical Society. d) The structure of the MEMS SAW gas sensor. e,f) Schematic illustrations of the working principle of the SAW gas sensor and frequency response curves of SnO_2_ quantum wires to NO_2_. Reproduced with permission.^[^ ^95^ ^]^ Copyright 2022, Elsevier. g) The structure of the MEMS QCM gas sensor. h,i) The overall design of the QCM gas detection system and gas‐sensing performance of ZnSe samples. Reproduced with permission.^[^ ^96^ ^]^ Copyright 2024, Wiley‐VCH. j) The structure of the MEMS FBAR gas sensor. k,l) The overall design of an FBAR sensor array (four devices) for vapor detection and its response characteristics. Reproduced with permission.^[^ ^98^ ^]^ Copyright 2020, American Chemical Society.

Currently, MEMS‐integrated mechanical gas sensors can be categorized into three main types: cantilever, SAW, and QCM gas sensors. Cantilever sensors detect gas molecules by sensitively capturing subtle changes in the bending degree or vibration frequency of cantilever beams, as shown in **Figure** **5**a. To realize trace molecule monitoring, the cantilever is usually fabricated with silicon and carbon‐related compounds via a top‐down bulk micromachining technique. The thickness of the cantilever can be reduced to a range of hundreds of nanometers to a few micrometers, enabling versatile gas molecule detection through surface modification with a gas‐sensitive layer. For example, Wu et al.^[^ ^93^ ^]^ developed a miniature dual‐resonance photoacoustic sensor, consisting of a small T‐type photoacoustic cell and a silicon cantilever beam. The design achieves double resonance of the acoustic signal. This system enables high‐sensitivity methane detection, with promising sensitivity and detection limits. Wang et al.^[^ ^94^ ^]^ developed a resonant microcantilever gas sensor functionalized with MoS_2_ nanosheets, which exhibited high sensitivity (13.6 Hz ppm^−1^), a low detection limit of 10 ppb, and good selectivity toward formaldehyde at room temperature (Figure 5b,c). SAW gas sensors utilize the impact of gas molecules on the propagation characteristics of surface acoustic waves, such as changes in velocity, attenuation, or phase, to capture information about gas molecules. For example, Zhou et al.^[^ ^95^ ^]^ employed facet engineering to regulate the facet‐dependent properties of metal oxides and synthesized SnO_2_ quantum wires with different (110) facet ratios, which were integrated into SAW sensors to optimize gas sensing performance. They found that the mass loading effect was the key mechanism for improving the gas sensitivity of SAW sensors (Figure 5e,f). QCM‐based MEMS gas sensors operate by detecting changes in the resonant frequency of a quartz crystal induced by the adsorption of gas molecules. As gas molecules adsorb onto the crystal surface, the frequency shifts, indicating mass change and allowing for precise gas detection. This compact sensor is suitable for real‐time monitoring in applications like environmental monitoring, industrial safety, and healthcare, thanks to its low power consumption and compatibility with microfabrication techniques. In 2024, Wang et al.^[^ ^96^ ^]^ utilized a QCM resonator to analyze H_2_ adsorption kinetics on Pt‐ZnSe MEMS sensors, revealing enhanced adsorption capacity (0.76 wt%) and fast desorption rates, confirming the role of electron‐deficient Se sites in optimizing H_2_ sensing (Figure 5h,i).

In contrast to electrochemical gas sensors, mechanical gas sensors operate independently of chemical reactions between gas molecules and sensitive layers for detection and identification. They offer advantages over electrochemical counterparts such as high sensitivity, long‐term stability, and integration portability. However, achieving such high precision in gas detection requires advanced fabrication techniques, which increase manufacturing complexity and costs, thereby limiting their widespread commercial adoption. Additionally, mechanical gas sensors are susceptible to environmental factors such as temperature, humidity, and pressure fluctuations, which can affect their performance. For example, SAW gas sensors experience reduced sensitivity with rising temperatures, while QCM gas sensors are sensitive to humidity changes, potentially compromising measurement accuracy and stability. Cantilever gas sensors may also face reduced accuracy due to environmental disturbances. To overcome these challenges and enhance gas identification capabilities, ongoing research in mechanical gas sensors emphasizes various technological advancements. For instance, QCM sensors improve molecular selectivity through the modification of selective adsorption layers on their surfaces, while SAW sensors benefit from advanced signal processing algorithms to enhance performance. In addition, cantilever sensors increasingly adopt array configurations integrating multiple beams, thereby strengthening their selective gas detection capabilities.

In addition to the aforementioned types of gas sensors, other mechanical structures, such as film bulk acoustic resonators (FBAR) and piezoelectric micromachined ultrasound transducers (PMUT), have also been explored for gas sensing applications.^[^ ^97^ ^]^ FBAR sensors operate based on the principle of acoustic wave propagation through thin films, where gas adsorption induces changes in mass loading and consequently shifts in resonance frequency, making them highly sensitive to minute gas concentrations (Figure 5j). Yan et al.^[^ ^98^ ^]^ developed an HKUST‐1‐based FBAR gas sensor employing a hybrid inorganic–organic surface modification strategy, which enhanced water stability while preserving high sensitivity. The device achieved ppm‐level detection performance, comparable to that of state‐of‐the‐art gravimetric resonator sensors (Figure 5k,l). In contrast, PMUT sensors utilize piezoelectric materials to generate and detect ultrasound waves. When exposed to gas molecules, these sensors detect changes in acoustic impedance or resonance frequency, providing an effective mechanism for gas detection. Both FBAR and PMUT sensors offer advantages in terms of miniaturization, high sensitivity, and compatibility with integrated circuits, making them promising candidates for next‐generation gas sensing technologies. Their mechanics‐based detection methods, coupled with advanced materials, open new opportunities for developing compact, efficient, and highly selective gas sensors suitable for a wide range of environmental monitoring, industrial safety, and healthcare applications.

In summary, recent MEMS gas sensing technologies can be broadly classified into electrochemical, optical, and mechanical types, each offering distinct advantages and facing specific limitations in key metrics such as size, selectivity, detection limit, and measurement range (**Table** **1**). Electrochemical sensors, with their simple structure, compact footprint, and low cost, enable highly sensitive detection of common gases, but their intrinsic selectivity is limited and often requires enhancement through surface functionalization or selective catalysts.^[^ ^99^, ^100^ ^]^ Optical MEMS sensors exploit characteristic absorption spectra of target gases to deliver excellent selectivity and rapid response, with the added benefits of non‐contact and remote operation in extreme environments such as high temperature, high pressure, or corrosive atmospheres.^[^ ^101^, ^102^ ^]^ However, their reliance on complex optical designs and light sources increases fabrication cost and hinders integration into portable, low‐power systems. Mechanical MEMS sensors offer ultra‐high sensitivity for trace gas detection.^[^ ^103^ ^]^ However, they are vulnerable to mechanical noise and thermal drift, while packaging and long‐term stability remain major challenges.

**Table 1:** Summary of key performance metrics for different types of gas sensors.

Sensor type	Material	Size	Selectivity (average response ratio)	Limit of detection (LOD)	Detection range	Refs.
Electrochemical	Pt‐modified Nb‐doped TiO_2_	0.05 cm^3^	12.3	50 ppm	50–1000 ppm	[104]
	Co_3_O_4_@ZnO	∼900 µm × 700 µm	>20.5	26 ppb	0.33–66 ppm	[105]
	PdPt/SnO_2_‐M	3 mm × 2 mm × 1 mm	> 5.58	50 ppb	50–2000 ppb	[106]
	Pd‐W_18_O_49_ NWs	1.5 × 1.5 mm^2^	H_2_: 20.9 NH_3_: 7.0	H_2_: 51.0 ppb NH_3_: 10.5 ppb	LOD–50 ppm	[107]
	Au‐decorated In_2_O_3_	3.1 mm × 3.1 mm	10	1 ppm	1–10 ppm	[108]
	Ga‐Doped In_2_O_3_	1.0 × 1.0 mm	17.875	50 ppb	1–200 ppm	[109]
	α‐Fe_2_O_3_@ZnO@ZIF‐8	1 × 1 mm^2^	>15	200 ppb	0.2–10 ppm	[110]
Optical	Ge‐OI	2.14 mm^2^	TS	0.627 ppm	50–200 ppm	[111]
	ScAlN	Ø1 mm × 4 cm	TS	100 ppm	100–5000 ppm	[112]
	PEI	20 µm × 20 µm	TS	40 ppm	40–1000 ppm	[113]
	PEI	1 cm^2^	TS	400 ppb	1.5 ppm–100 %vol	[114]
	ScAlN	500 µm × 500 µm	TS	25 ppm	25–5000 ppm	[115]
	LiTaO_3_	1 mm × 1 mm	TS	CH_4_: 63 ppm CO_2_: 2 ppm CO: 11 ppm	LOD–20 000 ppm	[116]
	——	Ø9 mm × 2 mm	TS	CH_4_: 25 ppm CO_2_: 9 ppm	LOD–3.6% vol	[117]
	LiTaO_3_	Not specified	TS	5.6 ppm	5.6–1000 ppm	[118]
	——	2.43 cm × 1.25 cm × 1.8 cm	TS	0.72 ppm	0.72–2000 ppm	[119]
Mechanical	Pd	1.8 × 1.6 mm^2^	Not specified	< 10 ppm	1–250 ppm	[21]
	Cu‐TCPP‐Cu MOF	0.125 cm^2^	>5	65 ppb	0.1–100 ppm	[120]
	PtRh/SnO_2_	3 × 2 × 1 mm	>6.645	1 ppm	1–25 ppm	[121]
	MIL‐101(Cr)	500 µm × 500 µm	2.226	51 ppm	51–1000 ppm	[122]
	GO‐SnO_2_	∼8 mm × 2.5 mm	∼4.286	40 ppb	40 ppb–1 ppm	[123]
	MnFeCoNiCu	0.2 cm × 0.3 cm	>3	30 ppb	10–500 ppb	[124]
	Pd	600 µm	∼74	50 ppm	0%–1% vol	[125]
	Pd/Cu	2.5 mm	∼6	7 ppm	0.1%–4.5% vol	[22]
	PtCu/In_2_O_3_	Not specified	∼20	50 ppb	0.5–50 ppm	[126]

Note:The average response ratio represents the mean of the target gas response divided by responses to various interfering gases such as CH_4_, NH_3_, NO_2_, CO, etc. Selectivity, described as “TS (Target Specific),” indicates negligible response to interfering gases.

### 3.4 Other types of MEMS Gas Sensors

Beyond conventional categories, several emerging sensing architectures, such as optical fiber sensors and photoacoustic sensors, show considerable potential for next‐generation gas detection. Optical fiber sensors detect gas concentrations by modulating the propagation behavior of light in optical fibers (such as refractive index, interference intensity, or absorption spectra).^[^ ^127^, ^128^ ^]^ They inherently offer electromagnetic shielding, low detection limits, high sensitivity, and long‐distance transmission capabilities. The introduction of optical fibers also helps mitigate issues such as large size, complex optical paths, and high costs associated with traditional optical gas sensors. Selectivity can be further enhanced through surface modifications, such as the introduction of functionalized nanomaterials (e.g., metal oxide nanoparticles, MOFs, etc.).^[^ ^129^ ^]^ However, optical fiber sensors are typically sensitive to temperature and humidity changes, and challenges remain in terms of overall device integration and mass production capabilities.^[^ ^130^ ^]^

**Figure 6:**
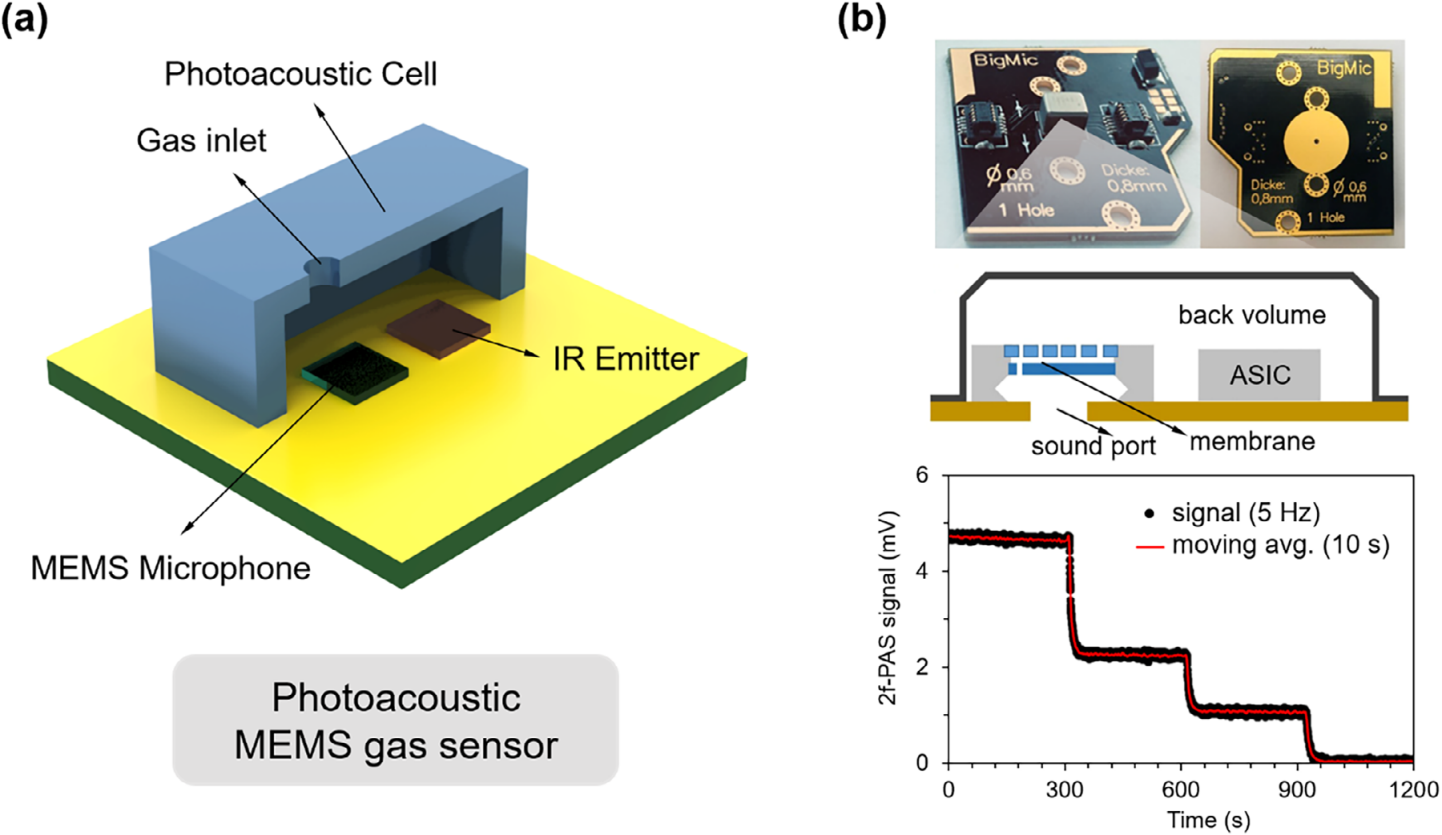
Structures and applications of photoacoustic MEMS gas sensors. a) Typical structure. b) Photograph of a bottom‐port MEMS mounted on a customized 0.8 mm‐thick PCB, with the sound port aligned to a 0.6 mm‐diameter hole on the board (top). Schematic diagram illustrating the MEMS microphone package with a bottom sound port (middle). 2f photoacoustic signal for stepwise CH_4_ concentrations (10, 5, 2.5, and 0 ppm in N_2_) recorded at 5 Hz (black line) and its 10 s moving average (red line) (bottom). Reproduced with permission.^[^ ^132^ ^]^ Copyright 2023, Elsevier.

Photoacoustic sensors comprehensively utilize light absorption and acoustic detection mechanisms: when target gases absorb periodically modulated light sources, non‐radiative relaxation effects occur, inducing local thermal expansion and thereby exciting acoustic signals, which can be detected by highly sensitive acoustic devices (such as MEMS microphones) (**Figure** **6**a).^[^ ^131^ ^]^ Thomas Strahl et al.^[^ ^132^ ^]^ developed a highly miniaturized and cost‐efficient MEMS photoacoustic methane sensor using an interband cascade laser, demonstrating ppb‐level sensitivity, excellent long‐term stability, and optimized acoustic resonance (Figure 6b). Compared to traditional NDIR sensors, photoacoustic technology does not require complex optical detectors and offers advantages in selectivity and sensitivity, with detection limits as low as the ppb level.^[^ ^133^ ^]^ In recent years, MEMS‐based integrated photoacoustic structures have gained increasing research attention, while still facing engineering challenges such as device miniaturization, selection of suitable light sources, and structural complexity.^[^ ^134^ ^]^ Overall, each type of gas sensor has its own strengths and limitations in performance, making it difficult to form absolute replacements. Targeted selection should be made based on the specific requirements of the application scenario.

## 4 Origin of Instability of MEMS Gas Sensors

The sensing film in a MEMS gas sensor is a thin material, typically composed of catalyst‐based nanomaterials or other chemically active media, designed for selective gas detection. However, instability in these films remains a major obstacle to achieving long‐term reliability and consistent performance, highlighting the importance of elucidating its underlying causes to guide the design of more durable sensing systems.

To quantitatively evaluate the long‐term stability and noise characteristics of MEMS gas sensors, especially those employing optical sensing mechanisms or requiring ultra‐low drift performance, Allan variance analysis is widely used. Originally developed in the field of frequency metrology and gyroscope signal evaluation, this method characterizes time‐domain signal fluctuations over varying integration times, effectively distinguishing between different types of noise (e.g., white noise, flicker noise, drift).^[^ ^135^ ^]^ In the context of gas sensors, Allan variance provides a statistical framework for identifying the optimal averaging time that minimizes measurement uncertainty while revealing the presence of long‐term drift or instability.^[^ ^136^, ^137^ ^]^ Incorporating such analysis has proven particularly valuable in benchmarking sensor performance for high‐precision and long‐term applications.

Here, we identify three principal mechanisms that contribute to the degradation of MEMS gas sensors: i) chemical degradation of the sensing film, reducing sensitivity and selectivity through irreversible surface reactions; ii) physical degradation of the film, where microstructural alterations impair gas–film interactions; and iii) deterioration of contact and heater elements, which undermines stable operation by disrupting thermal or electrical conditions. By systematically evaluating these factors, this work aims to inform strategies for extending the operational lifetime of MEMS gas sensors and guiding the development of next‐generation, stability‐optimized devices.

### 4.1 Chemical Degradation of the Sensing Film

Chemical degradation of the sensing film occurs when it reacts with chemicals in the surrounding environment, leading to deterioration or failure of the film material.^[^ ^138^ ^]^ This degradation can impact the physical and chemical properties of the sensing film, affecting the accuracy and sensitivity of the gas sensor.^[^ ^139^ ^]^ For instance, the film may weaken or lose its specific chemical reactivity, resulting in slower response times or erroneous readings.^[^ ^140^, ^141^ ^]^ Given the critical need for long‐term stability in chemiresistive sensors for various applications, we briefly summarize key factors related to chemical degradation of the sensing film, including changes in chemical structure, phase structure transformations, and surface poisoning.

#### 4.1.1 Chemical Structure Changes

The chemical stability of sensing films plays a pivotal role in maintaining the long‐term performance of gas sensors. However, under prolonged operation or harsh environmental exposure, the chemical structure of the sensing material can undergo significant transformations, leading to irreversible drift, reduced sensitivity, or even complete failure.^[^ ^142^, ^143^ ^]^ Among various degradation pathways, changes in the film's chemical structure are often triggered by external stimuli such as elevated temperature, exposure to high‐humidity environments, long‐term aging, and high‐energy radiation (such as ultraviolet light or ionizing radiation). These factors can induce surface reactions, phase transformations, bond breakage, or oxidation/reduction processes, ultimately altering the film's sensing properties. In the following sections, we discuss how each of these factors contributes to chemical changes in the sensing material.

##### Degradation under UV Light

**Figure 7:**
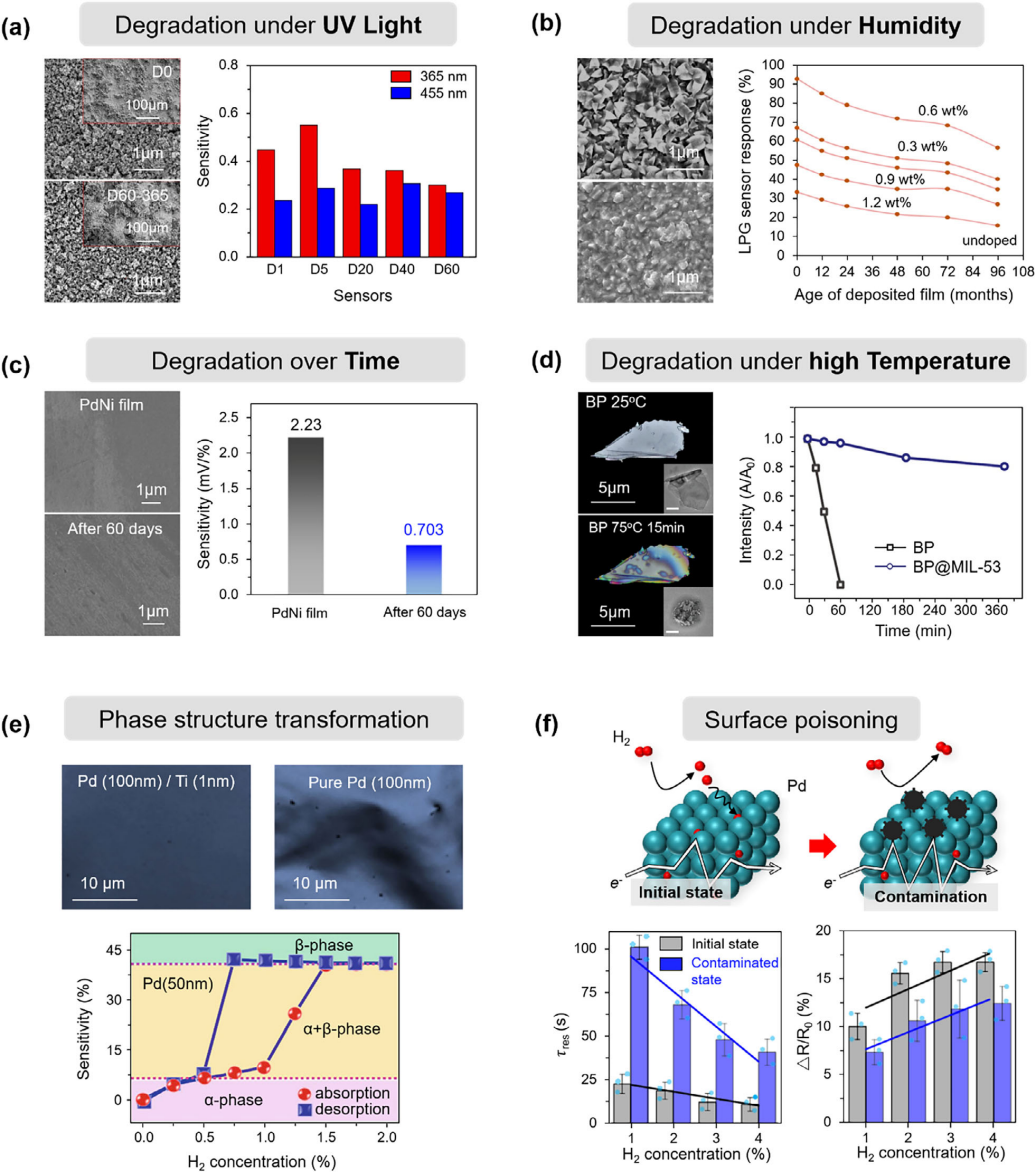
Chemical degradation of the sensing film. a) FIB‐SEM images of Ag‐ZnO nanoparticle‐based sensors before and after 60 days of NO_2_ exposure under UV light irradiation (365 nm, 40 mW cm^−2^) at room temperature, and sensitivity tests under the same irradiation conditions. Reproduced with permission.^[^ ^147^ ^]^ Copyright 2021, Elsevier. b) SEM micrographs of the MTO‐0.6(325) film before and after 96 months of exposure to a humid atmosphere, and the variation in LPG (1000 ppm) response with aging duration. Reproduced with permission.^[^ ^153^ ^]^ Copyright 2016, Elsevier. c) SEM images of the fresh PdNi film and the PdNi film after 60 days of exposure, and the sensitivity of the PdNi film sensor before and after 60 days. Reproduced with permission.^[^ ^169^ ^]^ Copyright 2024, American Chemical Society. d) Polarizing microscope and TEM images of BP before and after thermal treatment, and the variation in absorption ratios at 470 nm (*A*/*A*_0_) of BP. Reproduced with permission.^[^ ^186^ ^]^ Copyright 2020, Wiley‐VCH. e) Confocal laser scanning microscopy images of the surface morphologies of a pure Pd film and a Ti‐buffered Pd film after exposure to 2% H_2_, and the sensitivity changes of the pure Pd film as a function of H_2_ concentration. Reproduced with permission.^[^ ^192^ ^]^ Copyright 2011, Springer. f) Schematic illustration of degraded performance in a Pd hydrogen sensor due to surface contamination (black), along with a comparative analysis of response time and gas sensitivity in both the initial and contaminated conditions. Reproduced with permission.^[^ ^198^ ^]^ Copyright 2024, Springer Nature.

Conventional gas sensors typically rely on external heating sources to provide the activation energy required for gas‐sensing reactions. However, integrated heaters not only hinder device miniaturization but also compromise sensor stability during prolonged high‐temperature operation. Photoactivation technology has emerged as an effective alternative approach, with ultraviolet (UV) light sources—particularly those matching the bandgap of wide‐bandgap semiconductors—becoming a research focus in this field.^[^ ^144^ ^]^ Studies have demonstrated that appropriate UV irradiation can significantly enhance sensor sensitivity while reducing response/recovery times.^[^ ^145^ ^]^ Nevertheless, photoactivated devices inevitably experience performance degradation during long‐term operation, with this effect being particularly pronounced under UV illumination. The fundamental mechanism involves the disruption of chemical structural stability through continuous radiation exposure, manifesting as: lattice quality deterioration, increased surface defect density, abnormal elevation of oxygen vacancy concentration, and material degradation.^[^ ^146^ ^]^ Zhang et al.^[^ ^147^ ^]^ systematically investigated the performance degradation mechanisms in photoactivated room‐temperature NO_2_ sensors. TEM analysis (**Figure** **7**a) revealed a certain degree of agglomeration in the sensitive materials. Further XRD characterization confirmed significant deterioration in crystalline quality and a marked increase in oxygen vacancy defect concentration following multiple UV irradiation cycles. Notably, the performance degradation under visible light irradiation was considerably less pronounced compared to UV exposure. It is particularly noteworthy that UV‐induced performance degradation likely stems from synergistic effects with target gases rather than from radiation alone. In their study of UV‐activated In_2_O_3_ nanoparticle sensors, Ghorbani et al.^[^ ^148^ ^]^ observed that simultaneous exposure to UV radiation and formaldehyde (10–50 ppm) induced significant nanoparticle (NP) agglomeration, accompanied by compromised response stability and repeatability. These phenomena were not observed under either UV irradiation or formaldehyde exposure alone. This finding provides compelling evidence that the structural modifications result from cooperative interactions between UV radiation and target gas molecules.

In conclusion, these structural alterations not only diminish the gas‐sensing characteristics of devices but also severely impact long‐term operational stability and repeatability. Therefore, when developing UV‐activated gas sensors, it is imperative to thoroughly consider the long‐term effects of optical irradiation on material structures. Optimization of illumination parameters, including potential implementation of intermittent UV activation or visible‐light activation strategies, should be pursued to extend device operational lifetimes.

##### Degradation under Humidity

In practical applications of gas sensors, environmental humidity is a ubiquitous and non‐negligible key interference factor, particularly in high‐humidity application scenarios such as breath gas detection and environmental monitoring. High‐humidity environments not only alter the electron transport characteristics of sensors through physical adsorption but may also induce changes in the chemical structure of sensitive material surfaces, thereby significantly affecting sensor performance.^[^ ^149^ ^]^ Taking MOS gas sensors as a representative case, water molecules adsorbed on the surface of metal oxide sensing materials can react with oxygen vacancies to form hydroxyl groups.^[^ ^150^ ^]^ This chemical modification process not only alters the distribution of surface active sites but also interferes with the charge transfer process in gas‐sensing reactions, ultimately leading to reduced sensor sensitivity, deteriorated response characteristics, and diminished long‐term stability.^[^ ^151^, ^152^ ^]^ Research by Skariah et al.^[^ ^153^ ^]^ demonstrated that prolonged exposure to a high‐humidity environment induces significant structural evolution on the surface of Mg‐doped SnO_2_ thin films, accompanied by the formation of an adherent layer on the substrate (Figure 7b). Further analysis confirmed that the material surface reacts with water molecules, generating ‐OH groups and forming a SnO(OH)/Sn(OH) overlayer. This hydroxylated layer, with a thickness of approximately 20–35 nm, hinders oxygen adsorption and gas molecule diffusion, leading to a decline in sensor sensitivity. In addition to MOS gas sensors, porous materials such as emerging metal–organic frameworks (MOFs) in SAW, QCM, and cantilever‐based MEMS gas sensors also face long‐term stability issues under high‐humidity conditions.^[^ ^154^, ^155^, ^156^ ^]^ Taking QCM gas sensors as an example, although quartz crystals can maintain stable operation in harsh environments, the MOF layer is prone to degradation under high‐humidity conditions, leading to a significant decline in sensor performance. The degradation of MOFs can be primarily attributed to two factors: first, the breakage of metal–ligand bonds;^[^ ^157^ ^]^ second, MOFs formed in high‐humidity environments exhibit higher stability compared to pristine MOFs,^[^ ^158^ ^]^ and this transformation may induce structural changes, thereby affecting the sensor's response and stability.

In addition to the aforementioned chemical changes, the influence of humidity on gas sensors involves multiple physical mechanisms, including competitive adsorption effects,^[^ ^159^, ^160^, ^161^ ^]^ the formation of a water‐blocking film,^[^ ^162^, ^163^, ^164^ ^]^ and response drift.^[^ ^165^ ^]^ These mechanisms collectively contribute to reduced sensor response, slower response/recovery rates, and diminished long‐term stability.^[^ ^166^ ^]^ Particularly for gas sensors such as QCM and SAW, which are highly sensitive to surface mass changes, even minor humidity fluctuations may significantly affect their performance.^[^ ^167^ ^]^ This is because water vapor adsorption on the sensor surface alters its effective mass and viscoelastic properties, thereby inducing shifts in resonance frequency or acoustic wave propagation velocity, consequently markedly compromising the sensor's stability and repeatability. Therefore, in the design of high‐performance gas sensors, optimization for humidity interference resistance (e.g., surface hydrophobic modification, material doping regulation, or temperature–humidity compensation algorithms) should be a key consideration to ensure device reliability and stability in complex environments.

##### Degradation over Time

During prolonged operation, the sensing film of MEMS gas sensors is prone to chemical structure degradation, which significantly reduces both sensitivity and long‐term stability. Among various degradation mechanisms, oxidation of sensitive materials represents a typical failure mode. For metallic or alloy‐based sensitive materials, prolonged exposure to oxygen‐rich environments can induce oxidation of constituent elements, leading to substantial alterations in surface structure and electronic properties, thereby affecting the gas‐sensing response characteristics of the sensor.^[^ ^168^ ^]^ For instance, Jin et al.^[^ ^169^ ^]^ found that the stability of surface acoustic wave hydrogen sensors based on PdNi thin films significantly decreased during long‐term operation. Further characterization revealed that the PdNi films underwent surface oxidation after prolonged air exposure, with partial Pd being oxidized to PdO_x_ (Figure 7c). The formation of this oxide layer not only increased the adsorption/dissociation energy barrier for hydrogen on the Pd surface, inhibiting the formation of PdH_x_, but also reduced the overall conductivity of the PdNi film, ultimately leading to deterioration of hydrogen sensing performance. Similarly, metal chalcogenides (such as MoS_2_ and SnS) are susceptible to oxidation in ambient air, resulting in reduced surface active sites, gas adsorption, and reactivity, thereby degrading gas‐sensing performance.^[^ ^170^, ^171^, ^172^ ^]^

Another critical performance degradation mechanism involves the deactivation of metal catalyst nanoparticles. Noble metal catalysts such as Pt, Pd, and Au are typically loaded onto the surface of metal oxide sensing materials to enhance gas adsorption and catalytic reaction capabilities.^[^ ^173^ ^]^ However, due to the weak interaction between these metal particles and the support, under harsh operating conditions such as long‐term operation or elevated temperatures, atomic migration and particle agglomeration of the catalysts readily occur, leading to a significant reduction in specific surface area and consequent weakening of catalytic activity.^[^ ^174^, ^175^ ^]^ Furthermore, although some noble metal surfaces are coated with metal oxide semiconductors, they may still undergo oxidation reactions during prolonged high‐temperature exposure, further compromising their catalytic function and accelerating sensor performance degradation.^[^ ^176^ ^]^

##### Degradation under High Temperature

The sensing performance of MOS‐based gas sensors is highly dependent on the operating temperature. Although elevated temperatures enhance surface reaction activity, they also introduce a series of challenges. High temperatures limit the safety and applicability of these sensors for detecting flammable and explosive gases, while prolonged operation may cause thermal degradation in the sensitive membrane, significantly compromising the device's sensitivity and operational lifetime.^[^ ^177^, ^178^, ^179^ ^]^

First, sustained high‐temperature environments readily induce abnormal grain growth in metal oxides, which is one of the primary causes of long‐term output drift in sensors.^[^ ^180^ ^]^ Second, grain boundary diffusion and interparticle sintering effects at high temperatures markedly reduce the material's specific surface area and porosity, thereby weakening gas adsorption and reaction capabilities and leading to diminished sensitivity.^[^ ^181^, ^182^, ^183^, ^184^ ^]^ Particularly for nanocrystalline materials with smaller particle sizes (3—5 nm), structural reconstruction is more likely to occur at high temperatures, further degrading their thermal stability and sensing performance.^[^ ^185^ ^]^

Moreover, if the material exhibits poor thermal stability, thermal degradation may occur, resulting in performance deterioration or even complete sensor failure. For instance, Han et al.^[^ ^186^ ^]^ observed during thermal stability testing of black phosphorus that apparent surface bubbles emerged after merely 15 min of heating at 50 °C. As the temperature increased, both the number and size of bubbles further expanded, and after 15 min at 75 °C (Figure 7d), the destruction of the 2D layered structure was observed, causing the device's response to NO_2_ to vanish entirely. This phenomenon was attributed to the rapid degradation of black phosphorus at elevated temperatures. Similar issues exist for certain conductive polymer materials (e.g., polypyrrole (PPy) and polyaniline (PANI)), as their poor thermal stability undermines the long‐term stability of sensors under high‐temperature conditions.^[^ ^187^, ^188^ ^]^

For sensitive membranes constructed from amorphous metal oxide thin films, high temperatures may also induce crystallization processes, disrupting the structural homogeneity and integrity of the film and consequently affecting its electrical and gas‐sensing properties. In addition, for SAW gas sensors, variations in ambient temperature can induce drift in the resonant frequency of the device, primarily due to the temperature sensitivity of the acoustic wave propagation velocity in the piezoelectric substrate material.^[^ ^189^ ^]^ This thermally induced variation not only compromises the frequency stability of the sensor but may also reduce its detection sensitivity toward target gases. Furthermore, prolonged or cyclic exposure to high‐temperature operating conditions imposes more stringent requirements on the thermal stability of both the sensing layer material and the piezoelectric substrate.^[^ ^190^ ^]^ In the absence of appropriate material selection and temperature compensation mechanisms, the sensor performance may deteriorate significantly. The aforementioned degradation mechanisms collectively demonstrate that under high‐temperature operating conditions, sensitive materials and interfacial structures with superior thermal stability must be designed to achieve long‐term stability in sensor performance.

#### 4.1.2 Phase Structure Transformation

During the operation of gas sensors, the crystalline phase structure of sensing materials may undergo alterations due to high‐temperature environments or gas adsorption/desorption processes. Representative examples include: VO_2_ exhibits a structural transition from the monoclinic phase to the rutile phase under specific temperature conditions,^[^ ^191^ ^]^ while palladium thin films undergo reversible α‐to‐β phase transformations during hydrogen adsorption/desorption.^[^ ^192^ ^]^ Notably, such phase transition phenomena may significantly influence key sensor performance parameters, including sensitivity, selectivity, and response kinetics.^[^ ^193^ ^]^ These structural deformations may lead to sensor performance instability, becoming a critical factor affecting device reliability. To address this issue, Kim et al.^[^ ^192^ ^]^ proposed an effective strategy by introducing a Ti buffer layer between the Pd film and substrate. Experimental results demonstrated that this buffer structure not only suppressed excessive hydrogen diffusion in the Pd film and slowed the α‐to‐β phase transition but also enabled the device to exhibit excellent linear response and stable sensitivity across varying H_2_ concentrations, significantly enhancing the sensor's structural stability and response consistency (Figure 7e).

From the perspective of phase transition reversibility, the phase transformations of sensing materials can be categorized into reversible and irreversible types. Reversible phase transition materials can restore their initial crystalline structure upon removal of external stimuli without causing permanent physicochemical damage. This characteristic provides new opportunities for developing tunable gas sensors. For instance, Qin et al.^[^ ^194^ ^]^ fabricated a temperature‐controlled VO_2_ phase transition gas sensor via a one‐step solvothermal method, achieving reversible switching between the monoclinic and rutile phases of VO_2_ through precise temperature modulation, thereby dynamically tuning the sensor's selective response to acetone and ammonia. In contrast, irreversible phase transitions typically result in materials transforming into metastable or non‐equilibrium crystalline forms, with no spontaneous recovery to the initial structure.^[^ ^195^, ^196^ ^]^ Such irreversible changes often lead to performance degradation or even complete sensor failure. To ensure long‐term stability, unnecessary phase transitions should be prevented to enhance sensor durability.

#### 4.1.3 Surface Poisoning

During long‐term operation of gas sensors, surface poisoning emerges as one of the critical factors compromising their performance stability and response repeatability. Common poisoning sources include sulfur‐containing compounds (e.g., H_2_S, SO_2_), strongly oxidizing nitrogen oxides (e.g., NO_x_), and organosilicon compounds (e.g., HMDS).^[^ ^197^ ^]^ Under elevated temperatures or electrochemical reaction conditions, these substances readily undergo strong adsorption or even chemical reactions with the sensing materials, forming stable overlayers. Such surface overlayers severely hinder the effective contact between target gas molecules and active sites, leading to typical failure characteristics including significant sensitivity reduction, prolonged response time, deteriorated recovery capability, and potentially even false alarms. Kim et al.^[^ ^198^ ^]^ investigated the performance degradation mechanism of palladium nanowire sensors under prolonged exposure to ambient air, revealing that atmospheric CO_2_ forms C═O‐bonded contamination layers on the Pd surface, resulting in extended response time, reduced sensitivity, and long‐term stability deterioration (Figure 7f).

Notably, metal oxide semiconductor gas sensors exhibit particular susceptibility to surface poisoning effects.^[^ ^199^ ^]^ This primarily stems from their sensing mechanism's strong dependence on reversible adsorption‐desorption processes at active sites such as surface oxygen vacancies. Furthermore, noble metal catalysts (e.g., Pt, Pd, Au) widely employed in high‐performance gas sensors are equally vulnerable to toxic substances. These interfering gases can undergo strong adsorption on the catalyst surfaces or even form stable chemical bonds/new compounds, thereby occupying the active sites originally designated for catalytic reactions and significantly impairing their catalytic capability. Catalyst poisoning may also disrupt the oxygen species equilibrium on the sensing material surface, consequently affecting the electrical response characteristics of the entire sensing layer and ultimately leading to performance degradation and long‐term stability deterioration of gas sensors.^[^ ^200^ ^]^ Under harsh operating conditions, such as industrial emission monitoring or automotive exhaust analysis, poisoning resistance becomes critical. In high‐temperature, high‐humidity, or chemically complex gas environments, these effects can accumulate over time, severely compromising sensor reliability.

### 4.2 Physical Degradation of the Sensing Film

In addition to the chemical degradation of the sensing layer, physical degradation of the sensing film is also a critical factor affecting the long‐term stability of chemiresistive gas sensors. Chemiresistive gas sensors typically consist of a sensitive conductive layer and contacts, with their performance primarily determined by the sensing element, heating system, and substrate, among which the sensing element plays a central role.^[^ ^38^, ^201^ ^]^ Since these sensors operate in direct contact with the environment, their performance parameters tend to deteriorate over time. This is particularly evident under harsh environmental conditions, where frequent calibration is required to maintain accuracy.^[^ ^202^ ^]^ Changes in the microstructure of the sensing film, surface reactions/degradation of the film, and interfacial reactions with the substrate can all lead to sensor instability.^[^ ^203^ ^]^ Among various materials, semiconductor metal oxide thin films are the most extensively studied and widely applied sensing materials.^[^ ^204^ ^]^ Due to the fact that thermal energy promotes reactions between gas molecules and the surface of sensitive materials by overcoming activation energy barriers, most chemiresistive gas sensors operate at elevated temperatures.^[^ ^205^ ^]^ However, prolonged high‐temperature operation can negatively affect the sensing film, inducing thermal stress and mismatched thermal expansion between the film and substrate. These factors can lead to the formation of micro‐cracks within the film and delamination at the film–substrate interface. Such physical damage compromises the structural integrity of the film, reduces the adsorption and reaction efficiency of gas molecules, and causes signal drift in the sensor, ultimately degrading its sensitivity and long‐term stability.

#### 4.2.1 Crack Formation

**Figure 8:**
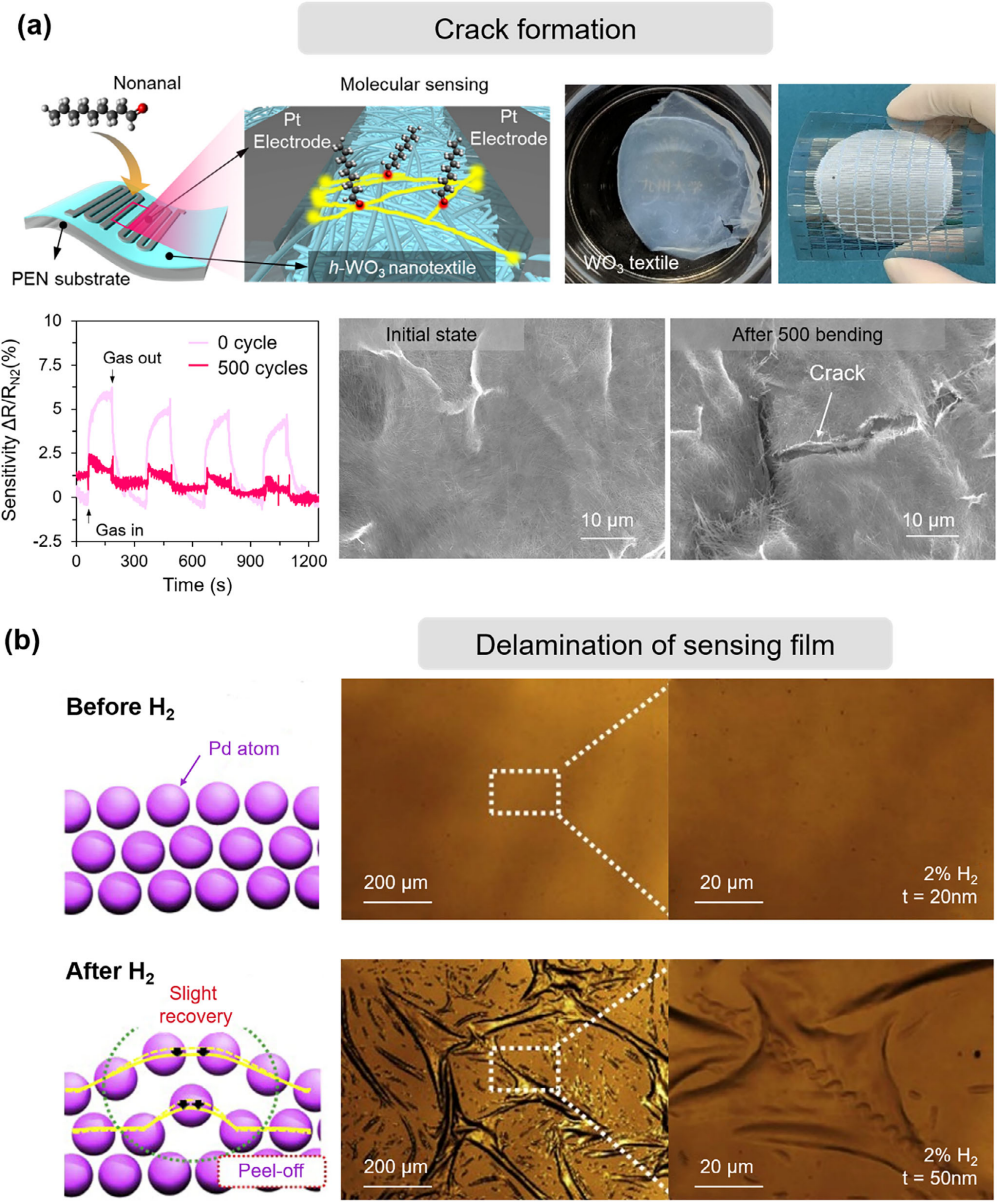
Physical degradation of the sensing film. a) Schematic illustrations of an *h*‐WO_3_ nanowire‐based molecular sensing device for lung cancer biomarker detection (upper), and its gas response to 2.67 ppm nonanal before and after mechanical bending at room temperature, along with SEM images of the sensor before and after 500 bending cycles (lower). Reproduced with permission.^[^ ^211^ ^]^ Copyright 2020, Royal Society of Chemistry. b) Schematics of the Pd–H system before and after exposure to H_2_ (left), and scanning laser confocal images of thin Pd films with thicknesses of 50 and 20 nm after exposure to 2% H_2_ (right). Reproduced with permission.^[^ ^222^ ^]^ Copyright 2010, Elsevier.

In recent years, flexible gas sensors have attracted significant attention in wearable and portable electronic devices due to their superior sensing performance and mechanical flexibility. Compared with conventional rigid gas sensors, flexible sensors can be directly attached to electronic device surfaces, textiles, or even human skin for real‐time gas monitoring. However, in practical applications, flexible devices are frequently subjected to mechanical deformations such as bending, stretching, or compression. Repeated mechanical stress can easily induce the initiation and propagation of microcracks in the sensing materials or flexible substrates.^[^ ^206^, ^207^ ^]^ The accumulation of these cracks may lead to unstable sensor output signals, decreased response sensitivity, and even device failure under extreme conditions.^[^ ^208^, ^209^ ^]^ Particularly in sensing films constructed from low‐dimensional nanomaterials (e.g., nanowires, nanosheets), cracks tend to propagate along grain boundaries or material interfaces, thereby altering the intrinsic electronic properties of the materials.^[^ ^210^ ^]^ Moreover, in high‐humidity or corrosive gas environments, cracks may accelerate interfacial aging and material delamination, further deteriorating sensor stability and lifespan. In a study on the flexible sensing performance of hexagonal tungsten oxide (*h*‐WO_3_) nanowires, Zhang et al.^[^ ^211^ ^]^ observed that devices with short nanowires (average ∼15 µm) exhibited significant cracking after 500 bending cycles, resulting in a rapidly decreasing response to nonanal. In contrast, devices with millimeter‐long nanowires (average ∼200 µm) maintained structural integrity due to an enhanced “textile effect,” showing no cracks even after 10^4^ bending cycles. SEM images clearly revealed fracture of the short nanowire network under mechanical stress, attributed to fewer interconnection points and consequent stress concentration. The long nanowires formed a textile‐like flexible network through three‐dimensional entanglement, effectively suppressing crack formation via stress dispersion and maintaining sensor stability (response decay <9%) (**Figure** **8**a). This finding provides important insights for nanostructure design in flexible sensors: the spatial entanglement characteristics of high‐aspect‐ratio nanowires can impart excellent mechanical robustness to devices.

Notably, cracking issues are not unique to flexible devices. Traditional MEMS gas sensors also frequently experience film cracking due to material stress mismatch. The primary cause lies in the significant difference in thermal expansion coefficients between the sensing film and substrate, leading to internal stress accumulation during high‐temperature deposition or thermal treatment processes, thereby inducing crack formation.^[^ ^212^, ^213^ ^]^ Through finite element simulation analysis, Filipovic et al.^[^ ^214^ ^]^ demonstrated that film stress primarily originates from residual stress during deposition and thermomechanical stress generated during cooling, both closely related to the material's thermal expansion coefficient, film thickness, and process temperature. Crack formation is particularly prevalent in thicker films, with the likelihood increasing as film thickness rises, eventually leading to cracking when exceeding a certain thickness threshold. The appearance of cracks disrupts film continuity, altering its conductivity and gas permeation pathways, thereby affecting sensing performance.^[^ ^215^, ^216^, ^217^ ^]^

Under high‐frequency thermal cycling conditions such as pulsed heating, crack formation becomes more frequent because rapid and non‐uniform temperature variations induce severe thermal stress fluctuations in the film, leading to continuous accumulation and propagation of microcracks and ultimately causing temporal drift in device response.^[^ ^218^, ^219^ ^]^ Although reducing film thickness to approach the Debye length of oxide materials can enhance response sensitivity, it also significantly compromises mechanical stability. Therefore, in the design of high‐performance gas sensors, a balance must be struck between sensitivity and structural reliability.

#### 4.2.2 Peel‐Off of Sensing Film

Similar to the mechanism of crack formation, thermal and mechanical stresses are primary factors contributing to the delamination of the sensitive layer. Additionally, the reduction in interfacial adhesion and volumetric changes induced by phase transitions in materials may also lead to thin‐film delamination.^[^ ^220^, ^221^ ^]^ For instance, in Pd‐based hydrogen sensors, when hydrogen atoms diffuse into the interstitial sites of the Pd lattice beyond the solubility limit, Pd undergoes a phase transition from the α‐phase to the β‐phase. As hydrogen further diffuses, the β‐phase nucleates and expands within the α‐matrix. During this phase transition, local defect structures or vacancies may form within PdH_x_, helping to accommodate the volumetric expansion induced by hydrogen absorption. However, the influx of hydrogen weakens the bonding strength within the Pd lattice, reducing the adhesion between the thin film and the substrate, ultimately leading to delamination (Figure 8b).^[^ ^222^ ^]^ Notably, significant delamination has been observed in Pd films with a thickness of 100 nm.

During heating, differences in the coefficients of thermal expansion between the thin film and the substrate or electrode materials can generate substantial interfacial stress, eventually resulting in structural delamination.^[^ ^223^ ^]^ This delamination not only weakens the mechanical strength of the film but may also cause electrical contact failure between the film and the electrode, significantly impairing sensing performance. For example, Khan et al.^[^ ^224^ ^]^ investigated the deflection of micro‐hotplate membranes caused by localized heating during device operation and found that severe film deformation induces significant interfacial shear stress between the nitride substrate and the deposited film, which may ultimately lead to delamination. Similarly, Korotcenkov et al.^[^ ^225^, ^226^ ^]^ fabricated SnO_2_ and In_2_O_3_ thin films on Si, SiO_2_, and Al_2_O_3_ substrates using the spray pyrolysis technique and observed the formation of a 10–20 nm transitional layer composed of randomly oriented crystallites at the film–substrate interface. The presence of this transition layer suggests that interfacial structural inhomogeneity may reduce adhesion, thereby compromising the stability of the thin film. Additionally, their study revealed that SnO_2_ films deposited on silicon substrates retained significant mechanical strain even after thermal treatment, indicating that this strain primarily originates from the mismatch in thermal expansion coefficients between the substrate and the deposited film.

The primary failure mechanisms of brittle films on flexible substrates include film cracking, groove formation, and delamination. Under mechanical bending, stress relaxation occurs through the initiation and propagation of cracks. These cracks in functional coatings typically develop well before the substrate fails, often initiating at surface defects that act as stress concentration sites. For instance, Ueda et al.^[^ ^227^ ^]^ prepared WO_3_ films on a silicon substrate featuring a cross‐shaped platinum electrode. Their findings indicated that irrespective of the deposition conditions, the WO_3_ films exhibited peeling from the substrate, suggesting weak adhesion.

Furthermore, conventional silicon‐based micromechanical substrates face challenges related to the stability and fatigue resistance of multilayer silicon oxide/silicon nitride thin films. Although these films are crucial for the long‐term stability of MEMS devices, their reliability in high‐temperature environments is relatively poor. For example, silicon nitride is susceptible to hydrolysis at elevated temperatures, which can degrade adhesion between the sensing layer and the film material. As the temperature increases, interactions between silicon nitride and the silicon substrate may weaken, resulting in thin‐film delamination.^[^ ^202^ ^]^ This delamination not only affects the structural integrity of the device but also compromises its resistance to vibration and mechanical shock, which are critical in practical applications. Additionally, the fabrication process of thin films plays a crucial role in determining their overall performance. For instance, films produced via electrodeposition may exhibit insufficient interfacial bonding strength, leading to poor adhesion and premature failure when subjected to mechanical stress, especially under cyclic loading or harsh environmental conditions.^[^ ^228^ ^]^

To address these challenges and enhance the reliability of silicon‐based MEMS systems, it is essential to conduct further investigation into film–substrate interfacial interactions and optimize both thin‐film materials and manufacturing processes. In the future, advancements in fabrication technologies may enable the development of thin‐film materials with superior mechanical stability and environmental adaptability, thereby expanding their applications in sensors, actuators, and other MEMS devices.

### 4.3 Degradation of the Integrated Components

In addition to changes in the physicochemical properties of the sensing film, the degradation of critical components (such as heaters, contact electrodes, and infrared light sources) also significantly impacts the long‐term stability of gas sensors. Currently, metal oxide semiconductor gas sensors typically operate at elevated temperatures ranging from 150 to 450 °C, relying on embedded heating elements. These heaters serve a dual purpose: they provide sufficient thermal energy to the sensitive layer to achieve optimal operating temperature and facilitate the desorption of gas molecules, thereby restoring the sensor to its initial state. Typically, the heaters are embedded in the gas sensor device as monolithic structures or MEMS, featuring several contacts on top that transmit the electrical signals generated by the sensitive layer in response to the target gas. However, chemical‐physical degradation over time can lead to the deterioration of both the heater and contacts, which may adversely affect the stability and parametric performance of the sensor.

**Figure 9:**
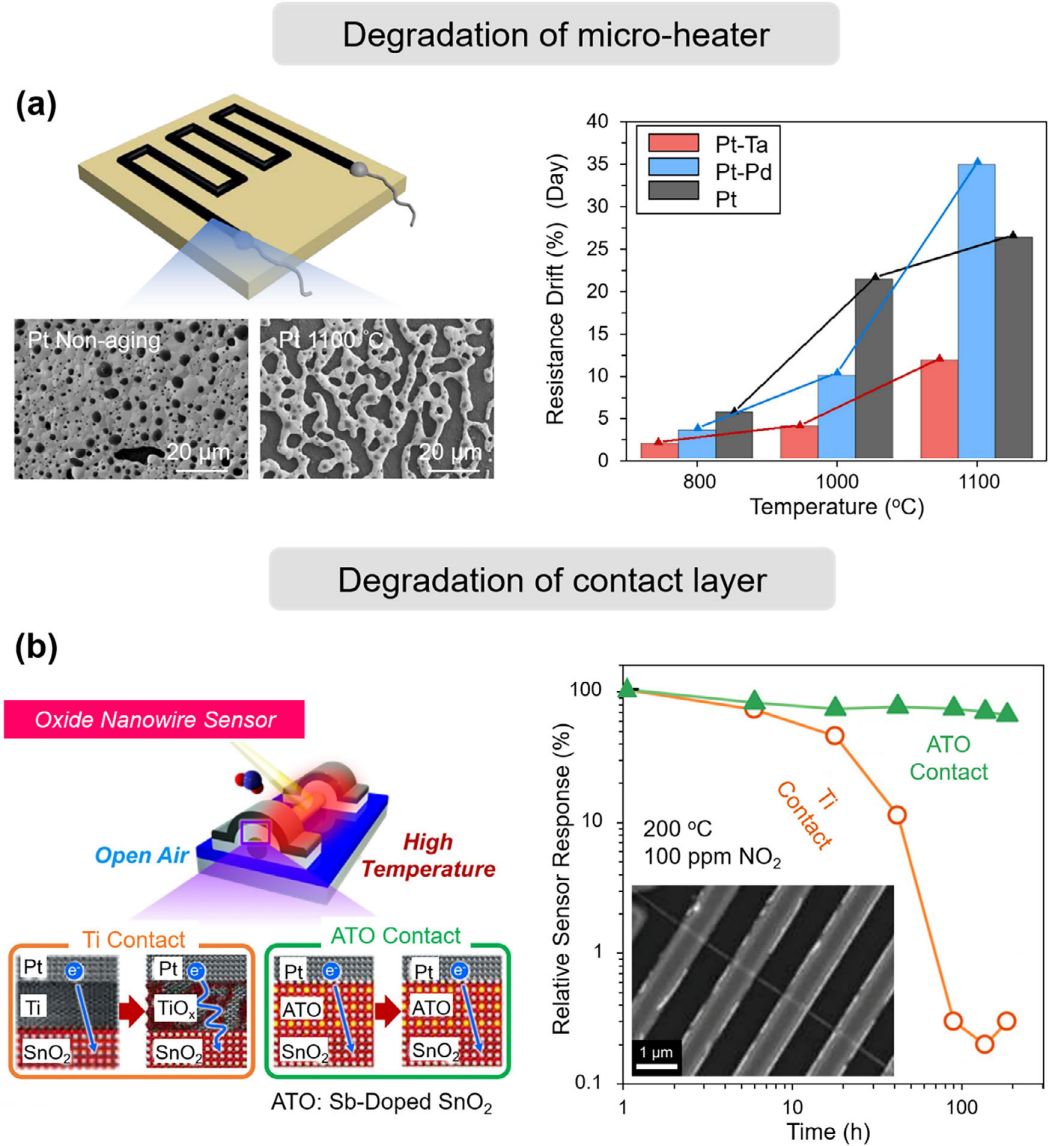
Degradation of the contact and heater. a) Structure of the Pt‐based microthermal plate and SEM images before and after thermal aging (left). High‐temperature resistance drift characteristics of Pt, Pt–Ta, and Pt–Pd system membranes at 800, 1000, and 1100 °C (right). Reproduced with permission.^[^ ^233^ ^]^ Copyright 2024, Elsevier. b) Structural changes of the conventional Ti contact and the proposed ATO contact after long‐term over extended periods (left). Relationship between the relative sensing response to 100 ppm NO_2_ (balanced with N_2_) and the duration of heating at 200 °C in open air (right). Reproduced with permission.^[^ ^235^ ^]^ Copyright 2017, American Chemical Society.

The choice of materials is a key determinant of microheater stability. Among the materials explored, polysilicon was initially widely adopted due to its compatibility with CMOS technology. However, polysilicon exhibits poor long‐term reliability, and its resistivity becomes unstable at temperatures exceeding 550 °C.^[^ ^229^, ^230^, ^231^ ^]^ To enable operation at higher temperatures, platinum has garnered significant attention. Pt offers superior chemical inertness, a stable temperature coefficient of resistance (TCR), excellent thermal conductivity, and a moderate coefficient of thermal expansion, making it more reliable than polysilicon under high‐temperature conditions.^[^ ^232^ ^]^ Unfortunately, platinum is not an ideal heating material due to several limitations. First, Pt exhibits poor adhesion to conventional silicon‐based substrates, necessitating the use of intermediate adhesion layers such as Ti or Ta. However, these adhesion layers tend to degrade at elevated temperatures. Second, Pt‐based heating materials are unsuitable for applications exceeding 1000 °C, as extreme temperatures induce severe degradation, including the formation of extensive voids within the Pt film, leading to significant resistance drift (**Figure** **9**a).^[^ ^233^ ^]^ This phenomenon arises from grain growth in Pt films, which increases diffusion and reduces stability at high temperatures. Additionally, electromigration under high‐temperature conditions promotes void formation within Pt grains, further exacerbating film degradation.

Molybdenum (Mo) has also been explored as a heating material, offering higher operational temperatures and improved stability compared to Pt. However, Mo is prone to oxidation and requires surface passivation for stable high‐temperature operation. Other materials, such as doped silicon, silicon carbide (SiC), and titanium nitride (TiN), have been investigated as potential alternatives for heating elements. Nevertheless, challenges such as low resistivity, poor contact performance, and electromigration issues persist. Consequently, Pt remains the dominant material for microheaters due to its superior overall performance.

In addition to the heater, the degradation of contact electrodes is another critical factor affecting sensor stability. As demonstrated by Choi et al.^[^ ^234^ ^]^ in their study on integrated graphene gas sensing channels, electrode contact degradation can significantly alter the resistance of the graphene channel, thereby deteriorating sensor performance. The degradation of contact electrodes typically originates from interfacial diffusion and oxidation reactions due to prolonged exposure to ambient conditions. For instance, Zeng et al.^[^ ^235^ ^]^ employed conventional Ti as the contact electrode for SnO_2_ nanowire sensors. Although the Ti electrodes initially exhibited good ohmic behavior and low contact resistance, they rapidly degraded within hours due to oxidation into TiO_x_ (Figure 9b), leading to unstable sensor performance. At elevated temperatures, conventional contact electrodes are even more prone to performance degradation, as high temperatures accelerate the aforementioned processes and may induce the formation of new phases while significantly increasing electromigration and thermomigration rates. This phenomenon fundamentally arises from the lack of thermodynamic equilibrium between the semiconductor substrate and the metal layer.^[^ ^236^ ^]^ Under high‐temperature operating conditions, in addition to material degradation, it is essential to consider the interplay among device power consumption, contact resistance, and heater stability. Elevated temperatures can lead to increased contact resistance, reduced thermal conduction efficiency, and localized overheating. To maintain the desired temperature of the sensing layer, the device must compensate with additional power input, which not only compromises energy efficiency but also exacerbates material degradation and shortens sensor lifespan. To mitigate these issues, selecting low‐resistance contact materials is crucial. Such materials can reduce localized overheating, enhance thermal conduction efficiency, lower overall power consumption, and delay device degradation, thereby improving sensor reliability and longevity in high‐temperature environments. Furthermore, an optimized thermal management design can contribute to stable sensor operation under harsh conditions.

Not only are chemiresistive gas sensors susceptible to degradation of critical functional components during long‐term operation, but optical gas sensors also face similar issues. For MEMS‐based NDIR gas sensors, the core infrared light source typically consists of a MEMS micro‐infrared emitter based on a micro‐thermal radiation structure. Such micro‐light sources inevitably experience aging effects during continuous operation, including gradual attenuation of radiation intensity and changes in spectral characteristics, which significantly impact the intensity and stability of infrared signals in the detection optical path, leading to reduced accuracy in analyzing the absorption features of target gases.^[^ ^237^, ^238^ ^]^ Particularly in MEMS structures, due to the small thermal capacity of the light source and its high sensitivity to environmental variations, the aging process is more likely to induce systematic signal drift, severely limiting the repeatability and long‐term stability of the sensor.^[^ ^239^ ^]^ Furthermore, optical components or gas detection chambers in practical applications are also prone to contamination by dust, aerosols, or condensates, further diminishing the sensor's sensitivity and introducing errors or drift in the output signal.^[^ ^240^ ^]^ In summary, the lifespan and stability of sensing components represent a critical bottleneck for achieving high long‐term stability in MEMS gas sensors.

## 5 Approaches used for Stability Improvement

### 5.1 Development of Chemically and Thermally Stable Sensing Films

The stability of gas sensors is influenced by multiple factors, among which the chemical stability and thermal stability of sensing materials are crucial.^[^ ^241^ ^]^ The higher the thermal stability of sensing materials, the more stable their operation can be maintained under high‐temperature or complex environments, particularly in the presence of reducing gases (such as H_2_ or CO).^[^ ^242^ ^]^ Chemical stability, on the other hand, determines the material's resistance to side reactions or interference from impurity gases in the environment, thereby enhancing the sensor's repeatability and long‐term reliability.^[^ ^243^ ^]^ The following section will discuss material design and fabrication strategies aimed at improving the thermal and chemical stability of gas sensors.

#### 5.1.1 Material Selection

To ensure the long‐term stable operation of MEMS gas sensors in practical applications, material selection must fully account for environmental factors, particularly in extreme conditions such as high temperature, high humidity, and corrosive gases. Materials with targeted resistance capabilities must be employed to prevent the induction of specific degradation mechanisms, which could otherwise weaken the device's performance and lifespan.

In high‐temperature environments (e.g., hydrogen production, combustion emission monitoring), sensing materials are prone to issues such as thermal stress accumulation, grain growth, phase transitions, or surface reconstruction, leading to response drift or failure. Therefore, materials with high thermal stability and low diffusivity should be prioritized. Traditional metal oxides (e.g., ZnO,^[^ ^244^ ^]^ SnO_2,_^[^ ^245^ ^]^ WO_3_^[^ ^246^ ^]^) typically remain stable only below 450 °C, losing sensitivity or undergoing structural changes at higher temperatures. For higher‐temperature applications, TiO_2_, with its excellent thermal stability and chemical inertness, emerges as a highly promising candidate material.^[^ ^247^, ^248^, ^249^ ^]^ Additionally, novel wide‐bandgap semiconductors such as SiC,^[^ ^250^, ^251^ ^]^ GaN,^[^ ^252^, ^253^ ^]^ and β‐Ga_2_O_3_^[^ ^254^, ^255^, ^256^ ^]^ exhibit high thermal conductivity, high electron mobility, and outstanding chemical stability, theoretically supporting operation at 600 °C or even close to 1000 °C. Notably, these materials have limited intrinsic surface activity and often require surface modification, noble metal loading, or heterojunction construction to enhance their gas‐sensing performance.^[^ ^257^, ^258^ ^]^ In acoustic devices like surface acoustic wave (SAW) and bulk acoustic wave (BAW) sensors, piezoelectric materials such as lanthanum gallium silicate (La_3_Ga_5_SiO_14_, LGS), yttrium calcium oxyborate (YCOB), and aluminum nitride (AlN) demonstrate excellent device compatibility at high temperatures due to their high phase transition temperatures and stable piezoelectric responses.^[^ ^259^, ^260^ ^]^

In high‐humidity scenarios (e.g., breath analysis, environmental monitoring), water vapor readily competes for surface active sites, leading to surface hydroxylation or even hydrolysis, thereby reducing sensitivity and causing long‐term performance drift. Studies indicate that, compared to n‐type metal oxides, p‐type semiconductor materials (e.g., NiO, CuO) generally exhibit superior humidity resistance.^[^ ^261^ ^]^ This may be attributed to their unique surface reaction mechanisms: the conductivity of p‐type materials is primarily governed by highly conductive hole accumulation layers, and their surface metal cations possess multiple oxidation states, facilitating strong oxygen adsorption and redox cycles, thereby inhibiting water molecule interference.^[^ ^152^ ^]^ Strategies to enhance humidity resistance mainly fall into two categories: (1) introducing hydrophilic components (e.g., NiO, CuO) to rapidly adsorb and regulate water behavior, preventing it from penetrating deep into the sensitive layer and interfering with detection; and (2) hydrophobic modification (e.g., using PDMS or rare‐earth oxides) to reduce the residence time of water molecules on the sensing film surface, thereby minimizing their interference with sensing behavior.^[^ ^262^, ^263^, ^264^ ^]^ Rare‐earth oxides such as CeO_2_, PrO_x_, and TbO_x_ are particularly noteworthy, as their mixed trivalent/tetravalent oxidation states enable reversible redox reactions to continuously remove surface hydroxyl groups, achieving “self‐healing” moisture desorption and significantly enhancing humidity stability.^[^ ^265^ ^]^

In industrial monitoring scenarios involving corrosive gases (e.g., NO_2_, SO_2_, H_2_S), sensors must contend with surface poisoning and structural decomposition caused by highly oxidizing or reducing gases. Thus, sensing materials must exhibit high chemical inertness and stable electronic structures. TiO_2_ is one of the most widely used corrosion‐resistant materials in such scenarios, particularly its rutile and anatase phases, which demonstrate excellent stability. Constructing p–n heterojunctions or doping with noble metals (e.g., Au, Pt) can further improve anti‐poisoning capabilities and gas response characteristics.^[^ ^266^, ^267^ ^]^ Other materials, such as MoS_2_,^[^ ^268^ ^]^ WO_3_,^[^ ^269^ ^]^ ZnO,^[^ ^270^ ^]^ and graphene derivatives, also exhibit strong corrosion resistance and multi‐gas compatibility.^[^ ^271^, ^272^ ^]^ Constructing composite structures or applying corrosion‐resistant coatings are common strategies to enhance corrosion resistance.

After systematically analyzing material selection strategies for extreme environments such as high temperature, high humidity, and corrosive gases, it becomes evident that while the degradation mechanisms triggered by these conditions differ significantly, they ultimately demand higher intrinsic structural and surface chemical stability from materials. In fact, even in most conventional applications with relatively mild environmental conditions, MEMS gas sensors still face challenges like physicochemical structural degradation and surface poisoning during long‐term operation. Therefore, even in more general working environments, material systems with high crystalline quality, low defect density, and excellent interfacial compatibility remain crucial to ensuring long‐term device stability and reproducibility. In this context, single‐crystal materials and two‐dimensional materials, owing to their unique crystal structures and outstanding physicochemical properties, have emerged as widely studied candidates for high‐stability materials.

##### Single‐Crystal Materials

**Figure 10:**
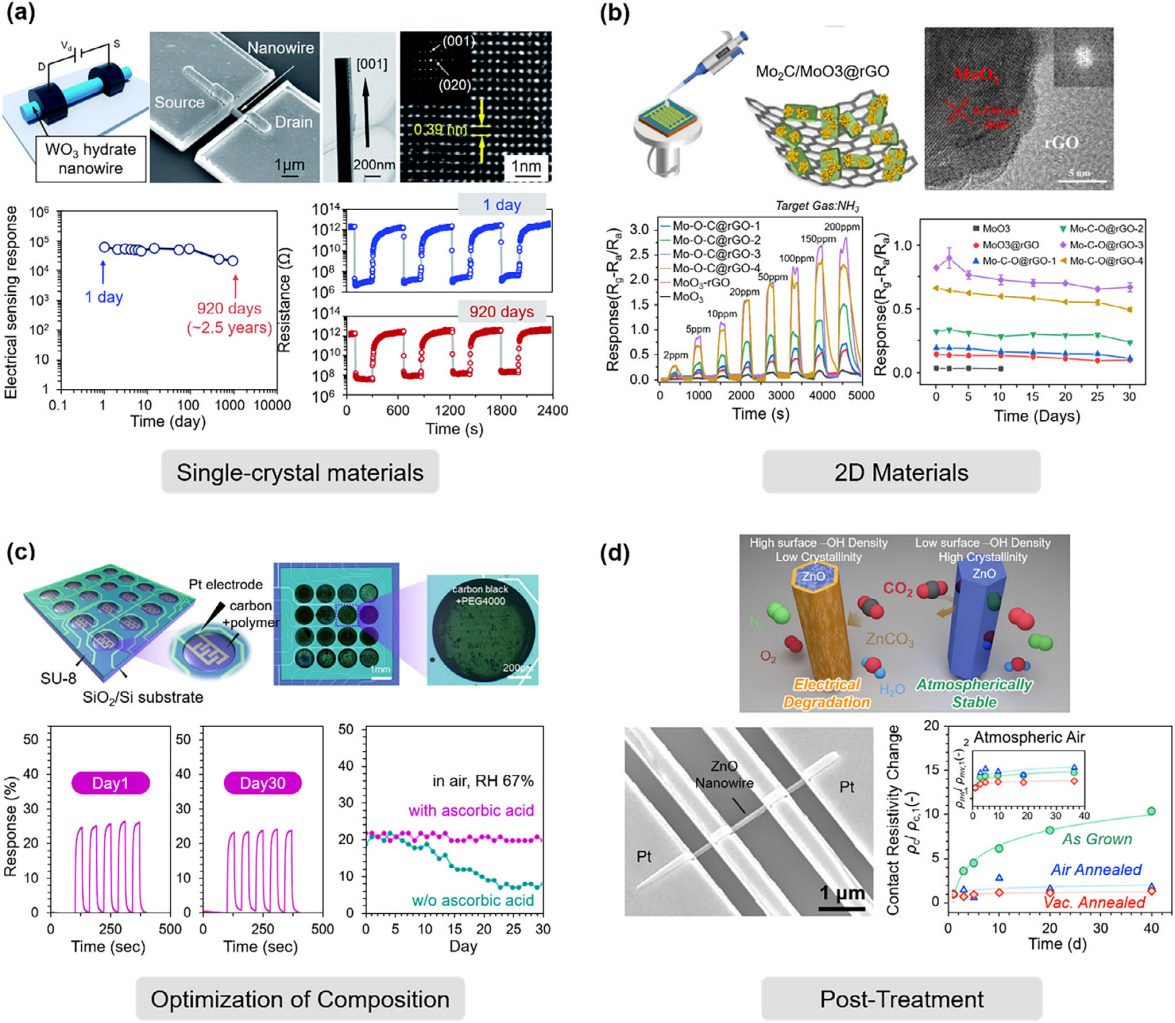
Development of chemically and thermally stable sensing materials. a) Schematic illustration and SEM image of a single WO_3_ hydrate nanowire sensor device (upper), along with TEM and SAED images of a WO_3_ hydrate nanowire. Long‐term stability of the sensor at 200 °C in response to 2.67 ppm nonanal, and dynamic resistance response after storage in air for 1 day and 920 days (lower). Reproduced with permission.^[^ ^275^ ^]^ Copyright 2021, Royal Society of Chemistry. b) Schematic diagram of the synthesis process of Mo_2_C/MoO_3_@rGO ternary composites, along with HRTEM image of MoO_3_@rGO (upper). Transient response curves of all sensors to 2–200 ppm NH_3_, and long‐term stability of the sensors to 5 ppm NH_3_ over a period of 30 days (lower). Reproduced with permission.^[^ ^287^ ^]^ Copyright 2023, American Chemical Society. c) Schematic illustration of the atmospheric electrical stability of ZnO nanowire devices (upper). FESEM image of a typical four‐terminal ZnO nanowire device (lower), along with the dependence of contact resistivity on ambient air preservation time for as‐grown, air‐annealed, and vacuum‐annealed ZnO nanowires. Nanowire resistivity, which correlates to contact resistivity, is shown in the inset. Reproduced with permission.^[^ ^307^ ^]^ Copyright 2019, American Chemical Society. d) Schematic illustrations and optical microscopy images of the chemiresistive sensor array (upper). Successive sensing responses to 2.7 ppm nonanal on Day 1 and Day 30, along with the response duration with and without the addition of AA (lower). Reproduced with permission.^[^ ^333^ ^]^ Copyright 2021, American Chemical Society.

Metal oxide semiconductor gas‐sensing materials have been widely used in gas detection due to their low cost, good stability, and high sensitivity. However, conventional MOS materials typically exist in polycrystalline forms, which inherently contain grain boundaries, surface defects, and non‐uniform active sites. These structural defects are detrimental to achieving repeatability and long‐term stability in sensors. In this context, single‐crystal materials with higher crystal quality and crystallinity have gradually attracted researchers' attention.^[^ ^273^, ^274^ ^]^ Compared with polycrystalline or amorphous materials, single‐crystal materials exhibit superior thermal stability and chemical durability, maintaining structural integrity and sensing performance even under high temperatures or corrosive atmospheres. This highly ordered structure reduces the likelihood of grain boundary migration and new defect formation, effectively preventing performance degradation. For instance, single‐crystalline SnO_2_, ZnO, and WO_3_ have been successfully applied in the detection of various gases, demonstrating excellent sensitivity and stability. Their highly ordered crystal structure not only enhances the material's intrinsic reliability but also provides an ideal platform for subsequent surface functionalization treatments. For example, Zhang et al.^[^ ^275^ ^]^ employed a strong acid treatment strategy to modify the surface of WO_3_·xH_2_O nanowires (**Figure** **10**a). By utilizing the strong hydration effect of SO_4_^2−^, they achieved dehydration of the nanowires at relatively low temperatures, inducing the formation of coordinatively unsaturated W sites on the surface. These active sites significantly enhanced the material's catalytic oxidation capability toward aldehyde molecules while reducing the desorption temperature of reaction products from 300 to 50 °C. Simultaneously, the system maintained thermal catalytic stability for over ten years at 300 °C. Further mechanistic studies revealed that reactant molecules could strongly adsorb onto these coordinatively unsaturated W sites, thereby preferentially initiating oxidation reactions and facilitating efficient product desorption. This strategy not only achieved synergistic optimization of molecular selectivity and long‐term stability but also demonstrated the great potential of single‐crystal structures in constructing stable, highly active sensing interfaces.

##### Two‐Dimensional Materials

Two‐dimensional (2D) materials have demonstrated unique advantages in gas sensing applications due to their high specific surface area, abundant surface active sites, and atomic‐level thickness.^[^ ^276^ ^]^ Their stable lattice structure and controllable defect density contribute to enhanced chemical selectivity and thermal stability of sensors. Currently, graphene and its derivatives, as well as transition metal dichalcogenides (TMDs, such as MoS_2_ and WS_2_), have exhibited excellent gas‐sensing performance.^[^ ^277^, ^278^, ^279^ ^]^ Among them, graphene, as a highly promising gas‐sensing material, features a two‐dimensional honeycomb lattice composed of sp^2^‐hybridized carbon atoms, ensuring that all atoms are exposed on the material surface, thereby enabling full interaction with target gases. TMDs possess a layered structure similar to graphene but exhibit semiconducting properties, including tunable band gaps and numerous active sites.^[^ ^280^, ^281^ ^]^ Additionally, TMDs demonstrate favorable room‐temperature gas‐sensing characteristics and have been widely employed in the detection of gases such as NH_3_ and NO_2_ at ambient conditions.^[^ ^282^, ^283^ ^]^

Despite these advantages, 2D materials still face certain limitations in terms of sensitivity and long‐term stability. To address these challenges, researchers often employ strategies such as heterostructure construction, doping modulation, defect engineering, and composite material design to further optimize their performance.^[^ ^284^, ^285^, ^286^ ^]^ For instance, Liu et al.^[^ ^287^ ^]^ developed a ternary composite material, Mo_2_C/MoO_3_@rGO, by integrating metal oxide (MoO_3_) with reduced graphene oxide (rGO) and introducing transition metal carbide (Mo_2_C), which exhibits excellent electrocatalytic properties (Figure 10b). The heterojunction formed in this ternary composite effectively modulates the interfacial band structure, optimizes charge carrier transport pathways, and enhances gas response signals. Mo_2_C, as a platinum‐like catalyst, possesses superior electrical conductivity and catalytic activity, facilitating improved reaction kinetics of NH_3_ molecules and promoting efficient desorption of reaction products. Meanwhile, rGO provides a continuous conductive network and abundant adsorption sites, not only enhancing charge transport but also accelerating gas diffusion. The synergistic effect among these three components significantly improves the response intensity, selectivity, and long‐term stability of the sensor toward NH_3_ at room temperature, broadening the application prospects of rGO‐based materials in room‐temperature gas sensing. In summary, 2D materials, owing to their structural tunability and functional versatility, are emerging as one of the most promising candidates for next‐generation high‐performance gas sensors.

#### 5.1.2 Design and Fabrication Strategies

In the optimization of sensor performance, the selection of sensitive membrane materials undoubtedly constitutes a core factor. However, the intrinsic advantages of materials can only be translated into high‐performance device‐level outputs when supported by rational structural design and fabrication processes. Therefore, after determining the material system, further unlocking its potential through microstructure design of the film, interface engineering, and precise control of fabrication techniques becomes a critical step in achieving gas sensors with high sensitivity, selectivity, and long‐term stability. The following section will discuss strategies for microstructural modulation of sensing films, device configuration optimization, and fabrication methods, aiming to provide systematic guidance for constructing high‐performance gas sensors.

Rational selection of film thickness serves as the foundation for optimized design. The thickness of the sensing film directly influences the sensor's sensitivity, response time, and mechanical stability.^[^ ^288^, ^289^ ^]^ Generally, the film thickness should be controlled within the range of several tens to hundreds of nanometers. Excessive thickness may lead to response delays and signal attenuation, while insufficient thickness can result in film delamination or damage due to poor adhesion.^[^ ^290^, ^291^, ^292^ ^]^ Thus, the film thickness should be adjusted according to the target gas type, sensor structure, and operating environment to achieve a balance between performance and stability. For metal oxide semiconductor‐based sensing films (such as SnO_2_, ZnO, etc.), the optimal film thickness is typically closely related to the material's Debye length. Generally, when the film thickness approaches or becomes smaller than the Debye length, the modulation of surface carriers induced by gas adsorption can affect the entire sensitive layer. This significantly enhances conductivity variations, thereby achieving higher response sensitivity.^[^ ^293^, ^294^ ^]^ Conversely, when the film thickness is much larger than the Debye length, the gas primarily interacts with the surface of the film, leaving deeper regions unable to effectively participate in the reaction, resulting in reduced sensing performance. However, this rule is not universally applicable. Variations in film thickness not only influence electrical behavior but also alter the film's porosity, surface roughness, and particle size distribution, structural factors that profoundly affect gas diffusion rates and adsorption capacity.^[^ ^295^, ^296^, ^297^ ^]^ Particularly for gases with larger molecular sizes, such as NO_2_ and VOCs, the film thickness range capable of forming highly porous nanostructures should be prioritized to ensure sufficient diffusion of gas molecules into the interior of the sensing film.^[^ ^298^ ^]^ Consequently, the gas‐sensing performance often exhibits a nonlinear dependence on film thickness. Generally, the sensing response intensity follows a trend of “first increasing and then decreasing” as the film thickness increases. The specific optimal film thickness should be experimentally determined and controlled within a reasonable range by considering the characteristics of the target gas, the properties of the sensitive material, and the operating environment of the sensor.^[^ ^299^, ^300^ ^]^ In practical fabrication, material dimensions and film thickness can be controlled by adjusting specific process parameters. For example, in the sol–gel method, appropriately reducing the precursor solution's pH can inhibit rapid hydrolysis and promote slower condensation reactions, leading to the formation of smaller, more uniformly distributed colloidal particles. This helps achieve dense, uniform, and thinner films. Increasing spin‐coating speed can also effectively reduce single‐layer film thickness. In vapor deposition processes (such as CVD and magnetron sputtering), parameters such as deposition time, gas flow rate, substrate temperature, working pressure, and target power can be finely tuned to precisely control film thickness and microstructure.

Additionally, the chemical composition of the film plays a crucial role in its performance. By carefully selecting materials, controlling component ratios, and employing high‐purity precursors, the selectivity toward target gases can be enhanced while suppressing background interference.^[^ ^301^, ^302^, ^303^ ^]^ Furthermore, the introduction of trace noble metal dopants (e.g., Pt, Pd, Au) can effectively improve the material's resistance to environmental temperature and humidity variations, thereby enhancing overall stability and reliability.^[^ ^304^, ^305^, ^306^ ^]^ To address the poor stability of polymer‐carbon nanocomposite gas sensors in ambient air, Li et al.^[^ ^307^ ^]^ proposed an improved strategy based on compositional optimization. Using a polyethylene glycol (PEG)‐carbon black (CB) model system, combined with synchronous infrared spectroscopy and electrical response measurements, the authors identified that the primary cause of sensor performance degradation was the oxidative consumption of PEG in air. Based on this mechanism, they introduced the antioxidant ascorbic acid (AA) into the sensing film to suppress PEG oxidation. Experimental results demonstrated that the modified sensor maintained stable responses for over 30 days in air (Figure 10c), significantly improving its long‐term stability. This strategy not only clarified the fundamental degradation mechanism but also provided a universal approach for optimizing the composition of sensing films, offering valuable insights for enhancing the stability of other polymer‐based sensors.

Selecting the appropriate preparation process is the key to achieving the aforementioned structural and compositional optimization. Existing preparation methods can be broadly categorized into three types: traditional wet chemical methods, vapor deposition methods, and emerging digital manufacturing technologies. Traditional wet chemical methods include sol–gel and self‐assembly techniques. The sol–gel method is a typical low‐temperature liquid‐phase preparation process widely used for depositing metal oxide‐based sensing films. Its principle involves the hydrolysis and polycondensation of metal–organic precursors to form a stable sol in the system, which is then deposited onto the substrate surface via spin‐coating, dip‐coating, or spray‐coating, followed by heat treatment to form a uniform nanostructured film.^[^ ^308^ ^]^ This method offers mild preparation conditions, simple processing, and low cost.^[^ ^309^ ^]^ However, it suffers from poor film uniformity and reproducibility, limited microstructure control, and susceptibility to multiple factors such as solvent, temperature, and precursor concentration, posing challenges for the consistent preparation of MEMS devices.^[^ ^310^ ^]^ In contrast, self‐assembly technology relies on intermolecular van der Waals forces, hydrogen bonds, or electrostatic interactions to arrange pre‐synthesized nanoparticles in an ordered manner on the substrate surface, enabling the construction of anisotropic structures and directional assembly.^[^ ^311^, ^312^ ^]^ It is commonly used to prepare sensing films with complex microstructures or specific orientations.^[^ ^313^ ^]^ Despite its excellent structural control capabilities, it still has limitations in large‐scale manufacturing and film stability.

Vapor deposition methods are currently the mainstream approach for preparing sensing films in MEMS gas sensors, primarily including chemical vapor deposition (CVD), plasma‐enhanced chemical vapor deposition (PECVD), magnetron sputtering, and atomic layer deposition (ALD). CVD is a technique that involves the chemical reaction of gaseous precursors at high temperatures to deposit solid materials, suitable for depositing dense and uniform metal oxides and metal sulfides as sensitive materials, particularly excelling in the preparation of two‐dimensional materials.^[^ ^314^ ^]^ Compared to traditional mechanical and liquid‐phase exfoliation methods, CVD can effectively control reaction kinetics and film morphology by adjusting process parameters such as temperature, reaction pressure, and carrier gas flow.^[^ ^315^, ^316^, ^317^ ^]^ PECVD, a variant of CVD, introduces plasma to excite reaction gases, enabling the deposition of high‐quality films at lower temperatures. This not only reduces the thermal stability requirements for substrates but also offers excellent film uniformity and microstructure control.^[^ ^318^, ^319^ ^]^ Magnetron sputtering is a typical physical vapor deposition method where high‐energy plasma bombards a target to sputter atoms or ions, which then deposit onto the substrate to form a film. It is commonly used to prepare high‐purity, dense metal oxides such as ZnO, SnO_2_, and WO_3_, widely applied in MEMS gas sensors. Atomic layer deposition (ALD) is a layer‐by‐layer deposition technique based on surface self‐limiting reactions, achieving atomic‐level thickness precision and excellent three‐dimensional coverage by alternately introducing precursor gases. It is particularly suitable for preparing high‐aspect‐ratio structures such as nanowires and nanotubes and has shown potential in high‐performance MEMS gas sensors.^[^ ^320^, ^321^, ^322^ ^]^ However, most vapor deposition methods require controlled atmospheres or vacuum conditions, involving complex equipment and high costs, which are factors to consider for large‐scale production.

In recent years, with the rapid development of digital manufacturing technologies, methods such as 3D printing and lithography have gradually been introduced into the preparation of gas sensors. As an emerging additive manufacturing technology, 3D printing eliminates the reliance on molds or masks in traditional processes, enabling the direct layer‐by‐layer construction of sensitive components on substrates.^[^ ^323^ ^]^ It demonstrates high flexibility and rapid prototyping capabilities, especially in the design of complex structures such as sensor microcavities, gas flow channels, and microheaters.^[^ ^324^, ^325^ ^]^ However, it currently faces challenges in achieving integrated printing of entire MEMS sensor structures, and the coexistence of multiple materials poses risks of cross‐contamination, making process integration still challenging.^[^ ^326^ ^]^ Lithography, as a core patterning transfer method in the microelectronics industry, has been widely applied in the precision machining of microstructures for MEMS gas sensors. Its basic principle involves using a photomask to transfer the desired pattern onto a photoresist layer via UV exposure, followed by development and etching steps to achieve high‐precision pattern transfer on the substrate or functional material surface.^[^ ^327^ ^]^ This technology can be used not only for preparing micro‐scale device components such as metal electrodes, heaters, and microcavities but also for the patterned preparation of sensitive materials, making it particularly suitable for high‐consistency batch production of array devices.^[^ ^328^, ^329^ ^]^ Building on traditional mask lithography, advanced patterning methods such as deep ultraviolet (DUV) lithography and electron beam (e‐beam) lithography further enhance pattern resolution and structural complexity.^[^ ^330^ ^]^ Lithography not only plays a crucial role in improving device structure consistency and array repeatability but also provides an important platform for multi‐material integration and micro‐nanostructure control, serving as a key supporting technology for the scalable manufacturing and performance enhancement of MEMS gas sensors.^[^ ^331^ ^]^

In addition to the rational design and fabrication strategies discussed above, post‐treatment approaches can further enhance the structural stability of sensing films in complex environments and ensure consistent gas‐sensing responses. For instance, thermal annealing and UV/ozone treatment can be employed to suppress environmentally induced aging reactions, surface poisoning, and performance drift.^[^ ^332^ ^]^ For instance, addressing the electrical instability of ZnO nanowire devices in ambient air, Nakamura et al.^[^ ^333^ ^]^ proposed a material optimization strategy centered on thermal treatment. Heat treatment of ZnO nanowires not only significantly improved crystallinity while reducing grain boundary and defect concentrations, but also effectively diminished surface hydroxyl (−OH) groups, thereby reducing reactive sites for CO_2_ adsorption. The enhanced crystal quality reinforced structural stability, while the reduction of hydroxyl groups inhibited carbonate layer formation. Ultimately, this strategy enabled the device to maintain electrical stability for at least 40 days in air (Figure 10d), validating the critical role of thermal treatment in improving the long‐term stability of sensing materials. This approach provides reliable material design and process optimization insights for constructing highly stable gas‐sensing elements.

Finally, even when the sensing film itself exhibits excellent stability, prolonged exposure to humid, high‐temperature, corrosive, or dusty environments may still lead to performance degradation due to aging or inadequate encapsulation. Therefore, selecting suitable encapsulation materials and methods is equally critical. Ideal encapsulation materials should possess superior hermeticity, corrosion resistance, and environmental adaptability to effectively block the intrusion of external harmful gases and moisture, thereby preventing interference with the sensing film and internal components.^[^ ^334^ ^]^ High‐quality encapsulation solutions not only enhance the sensor's resistance to mechanical shock and chemical corrosion but also extend device lifespan, improve reliability, and potentially reduce manufacturing costs.^[^ ^335^, ^336^, ^337^, ^338^ ^]^ It is important to note that different application environments impose varying encapsulation requirements. Thus, the most suitable encapsulation materials and technologies should be carefully matched to specific operational scenarios to expand the applicability and potential of gas sensors.

### 5.2 Development of Room Temperature Gas Sensors

In order to overcome the activation energy barrier required for surface reactions and enhance the rate of surface reactions in gas‐sensitive materials, while improving the sensor's responsiveness and reversibility, it is usually necessary to provide sufficient thermal energy. Consequently, conventional metal oxide gas sensors often require operation at elevated temperatures ranging from 150 to 500 °C.^[^ ^339^, ^340^, ^341^, ^342^, ^343^, ^344^ ^]^ However, high‐temperature operating conditions can readily induce thermal‐driven grain growth, thereby affecting gas diffusion behavior at grain boundaries. Furthermore, sintering effects may lead to undesirable long‐term drift issues, ultimately compromising sensor stability and operational lifetime.^[^ ^345^, ^346^ ^]^ Additionally, when detecting flammable gases, elevated temperatures may pose explosion risks.^[^ ^347^, ^348^ ^]^ Therefore, the development of gas sensors capable of stable operation at room temperature holds significant importance for enhancing device safety and long‐term stability.^[^ ^349^, ^350^ ^]^ To achieve this objective, researchers have proposed various strategies to improve material gas‐sensing performance at ambient temperature, including noble metal loading and photoactivation approaches.^[^ ^351^, ^352^, ^353^, ^354^, ^355^ ^]^ Table 1 provides a summary of recent studies on the development of room‐temperature sensors employing these two strategies.

#### 5.2.1 Loading Noble Metal

**Figure 11:**
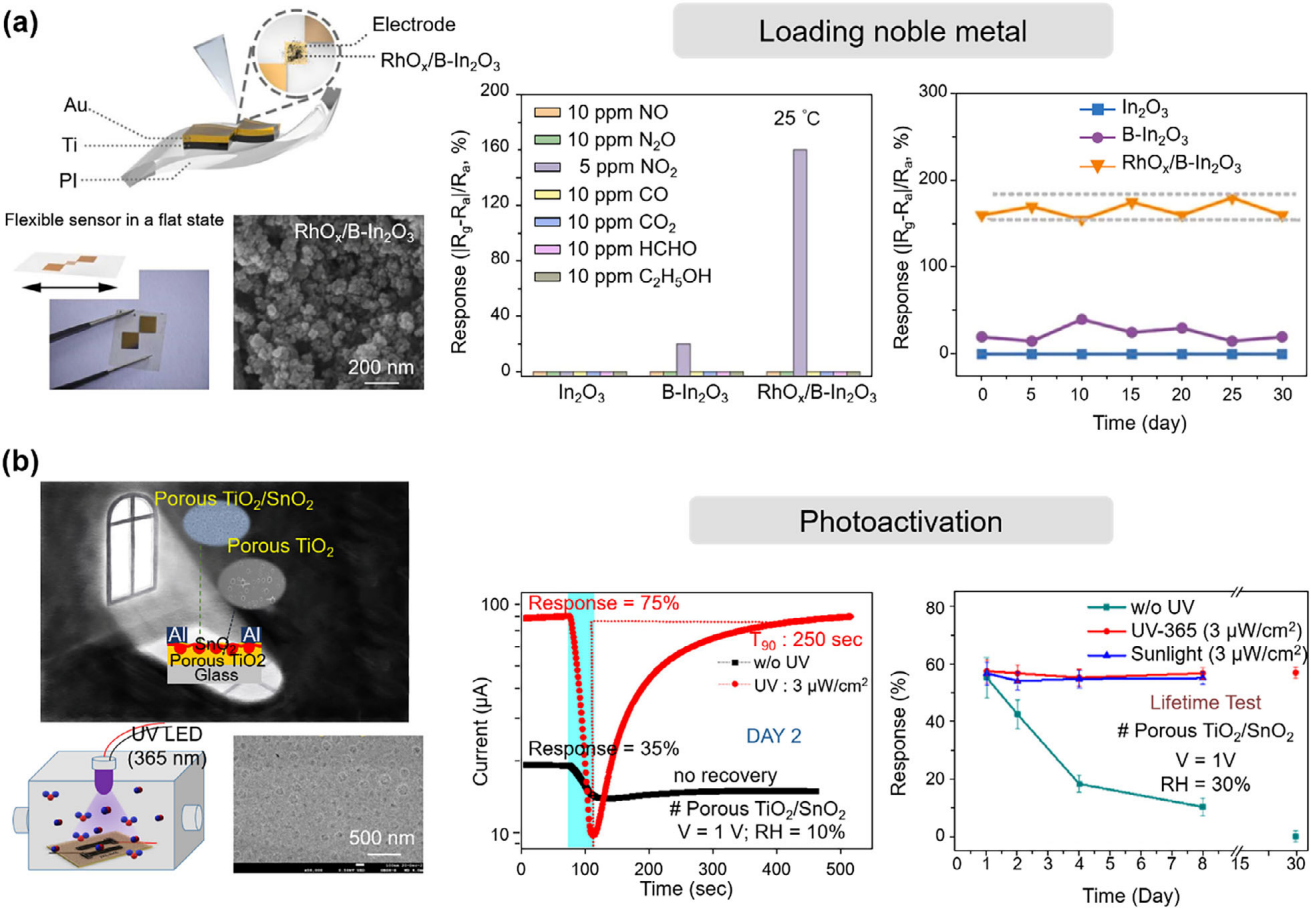
Development of room temperature gas sensors. a) Schematic illustration and application of the flexible sensor structure, along with an SEM image of RhO_x_/B‐In_2_O_3_ (left). Selectivity and long‐term stability comparisons of In_2_O_3_, B‐In_2_O_3_, and RhO_x_/B‐In_2_O_3_ gas sensors (right). Reproduced with permission.^[^ ^356^ ^]^ Copyright 2024, Elsevier. b) Schematic illustrations and SEM image of the UV‐activated porous TiO_2_/SnO_2_‐based sensor for NO detection at 4 ppb at room temperature (left). Gas response on Day 2 under UV and dark conditions, and long‐term performance toward 500 ppb NO under dark, UV illumination, and sunlight after 8 and 30 days (right). Reproduced under the terms of the CC BY 4.0 license.^[^ ^361^ ^]^ Copyright 2025, American Chemical Society.

The deposition of noble metals on semiconductor metal oxide surfaces represents an effective approach for achieving room‐temperature gas detection. The enhancement effect of noble metals on gas‐sensing performance primarily involves chemical and electronic mechanisms. The chemical effect can be explained through the “spillover effect”: noble metal nanoparticles serve as highly efficient catalysts that promote the adsorption and dissociation of oxygen molecules, subsequently migrating the generated active oxygen species to the metal oxide surface, thereby increasing the concentration of surface‐active oxygen. Additionally, analyte gases can also preferentially adsorb onto the surface of noble metals, where they dissociate under catalytic action and subsequently migrate to the oxide surface to participate in the reaction. This process accelerates electron release and transfer, significantly improving the reaction efficiency between gases and materials. The electrical effect originates from the work function difference between the noble metal and the metal oxide, which drives electrons to migrate from the oxide to the noble metal, leading to the formation of a Schottky barrier and an additional depletion layer. This barrier modulates charge carrier transport behavior at the interface, thereby enhancing sensor sensitivity. Furthermore, noble metals can improve electron transfer rates at oxide surfaces, further boosting response speed. For instance, Wei et al.^[^ ^356^ ^]^ employed a self‐assembly method to deposit amorphous RhO_x_ on B‐In_2_O_3_ surfaces, forming RhO_x_/B‐In_2_O_3_ composites. Through electronic and chemical sensitization effects, RhO_x_ significantly improved the gas‐sensing performance of B‐In_2_O_3_ by enhancing surface‐adsorbed oxygen concentration and reducing reaction activation energy, thereby improving gas response, selectivity, and reaction/recovery times. Compared with pure B‐In_2_O_3_ sensors, the RhO_x_/B‐In_2_O_3_ sensors demonstrated higher response, faster recovery, and better stability, capable of detecting NO_2_ concentrations as low as 1 ppm (**Figure** **11**a). Similarly, Nair et al.^[^ ^357^ ^]^ designed a bimetallic‐loaded carbon nanofiber hydrogen sensor that utilized the synergistic catalytic effects of gold and platinum to significantly enhance hydrogen adsorption and reaction activity, thereby improving response speed and sensitivity. The incorporation of noble metal nanoparticles into composite materials can further enhance gas‐sensing performance. By introducing additional active sites and reducing the activation energy of gas‐sensing reactions, noble metals significantly promote rapid gas molecule adsorption and reaction, enabling efficient detection at lower temperatures.

It is worth noting that, although noble metal doping is an established strategy for enhancing the gas‐sensing performance of metal oxide semiconductors, its effectiveness is highly selective, and not all noble metals confer optimal modification benefits. Research indicates that the degree of sensing performance improvement primarily depends on three key factors: the intrinsic properties of the noble metal (e.g., catalytic activity and electronic structure), its chemical state within the material (metallic or oxidized), and microstructural characteristics (including dispersion and particle size distribution). More importantly, whether effective synergy can be established between noble metals and base materials often determines the extent of gas‐sensing performance enhancement. Particularly, the work function difference between noble metals and semiconductors plays a crucial role in modulating interfacial electron transport behavior: when the noble metal's work function is lower than that of the semiconductor, electrons tend to transfer from the noble metal to the semiconductor surface, increasing the number of electrons captured by adsorbed oxygen and expanding the depletion layer, thereby enhancing responses to reducing gases. Conversely, when the noble metal's work function (especially in oxide form) exceeds that of the semiconductor, electrons may flow from the semiconductor to the noble metal, reducing surface‐active oxygen species and weakening electronic sensitization effects. In such cases, noble metals are more likely to promote target gas molecule dissociation or oxidation reactions through their superior catalytic properties, manifesting as chemical sensitization effects.^[^ ^358^ ^]^ Therefore, different noble metal dopants may operate through distinct enhancement mechanisms, with the final effect depending on the noble metal's electronic structure, surface chemical properties, and interactions with semiconductor materials, necessitating careful selection and optimization for specific material systems.

#### 5.2.2 Photoactivation

Photoactivation is one of the most important strategies for achieving high‐performance gas sensing at room temperature, as it can significantly enhance key performance metrics such as sensitivity, response/recovery speed, reversibility, and humidity resistance. Typical photoactivation methods include ultraviolet and visible light activation. Taking UV activation as an example, when the energy of incident photons is greater than or equal to the material's bandgap, electrons in the valence band can be excited to the conduction band, generating abundant photogenerated electron‐hole pairs.^[^ ^359^ ^]^ These photogenerated electrons can enhance the surface adsorption of oxygen molecules and promote the formation of reactive oxygen species such as O_2_^−^ (as shown in Equation 1), thereby significantly improving gas reaction activity, sensitivity, and response/recovery speed.^[^ ^360^ ^]^ Deb et al.^[^ ^361^ ^]^ developed a novel nanoporous TiO_2_/SnO_2_ heterojunction NO_x_ gas sensor capable of highly sensitive detection of 4 ppb‐level NO_x_ under only 3 µW cm^−2^ UV illumination. During the sensing process, UV light not only excites TiO_2_ to generate photogenerated electrons that are injected into the conduction band of SnO_2_, thereby enhancing the device's response performance, but also helps maintain current stability, accelerate response/recovery processes, reduce humidity interference, and extend the sensor's operational lifespan (Figure 11b). Further experimental results demonstrated that natural sunlight could replace UV LEDs as the light source, enabling low‐power, long‐term stable detection. Although most studies employ continuous UV irradiation to maintain device response, prolonged exposure may lead to decreased response performance, slower recovery rates, and consequently, reduced stability and increased power consumption. In contrast, intermittent UV irradiation exhibits weaker resistance modulation effects and less impact on the sensing film structure, resulting in superior device stability.^[^ ^362^, ^363^, ^364^, ^365^ ^]^

(1) $$\begin{array}{c} \mathrm{hv}\to h^{+}+e^{-}\\ O_{2}+{e^{-}}_{\left(\mathrm{hv}\right)}\to O_{2\left(\mathrm{hv}\right)}^{-} \end{array}$$

Notably, in some cases, the gas response induced by photoactivation is lower than expected, primarily due to the rapid recombination of photogenerated electron‐hole pairs. Constructing heterojunction structures can effectively suppress this recombination process, prolong the lifetime of photogenerated carriers, and thereby enhance photoactivation effects.^[^ ^366^, ^367^ ^]^ Additionally, loading noble metal nanoparticles can extend the material's light absorption range to the visible region.^[^ ^368^ ^]^ Zhan et al.^[^ ^369^ ^]^ investigated the influence of silver (Ag) loading on photoactivated gas sensing performance and found that light of different wavelengths could enhance the sensitivity of Ag‐loaded materials through the surface plasmon resonance (SPR) effect. Wang et al.^[^ ^370^ ^]^ fabricated a ZnO/Au room‐temperature NO_2_ and NH_3_ sensor that exhibited high selectivity toward NO_2_ under visible light irradiation, attributed to the SPR‐induced photogenerated electrons on Au nanoparticles promoting NO_2_ adsorption and desorption behaviors.

Despite the significant performance improvements that photoactivation technology can bring to metal oxide gas sensors at room temperature, several challenges remain, including the increased device volume and manufacturing costs due to external light sources and optical power monitoring systems, as well as interference from natural light in practical applications.^[^ ^371^ ^]^ Furthermore, Wang et al.^[^ ^372^ ^]^ reported that the response and recovery performance of a WO_3_ gas sensor showed no improvement under UV irradiation, indicating that the mechanisms of photoactivation effects vary among different metal oxide materials, and their efficacy in enhancing gas sensing performance depends on the material's band structure, surface states, and light absorption characteristics. Therefore, not all metal oxides exhibit excellent room‐temperature sensing performance toward target gases under UV excitation, and the effectiveness of photoactivation strategies requires systematic evaluation based on specific material systems (**Table** **2**).

**Table 2:** Recent research on room temperature gas sensors.

Methods	Material	Analysis	Concentration range	Operating temperature	Ref.
Loading noble metal	Pd/TiO_2_	hydrogen	lower than 20 ppm	RT	[373]
	Au decorated ZnO	CO	50 ppm	35 °C	[374]
	Pd‐loaded SnO_2_/PRGO	CO	50–1600 ppm	26 °C	[375]
	Pt–In_2_O_3_	NO_2_	ppb‐level	RT	[376]
	Rh‐doped SnO_2_ (PANI coated)	NH_3_	13.6–100 ppm	RT	[377]
	CNF@Ni–Pt	H_2_	0.01‐4%	RT	[357]
	Pd/ZnO	H_2_	LOD: ∼2.8 ppm	RT	[378]
	Ru‐CeO_2_	formaldehyde	LOD: 10 ppb	RT	[379]
	Pt@SnS2/MXene	NH_3_	23 ppb–10 ppm	RT	[380]
	Pt/TiO_2_	H_2_	3–10 000 ppm	RT	[381]
	Au NPs decorated In_2_O_3_/SnIn_4_S_8_	NO_2_	7–155 ppm	RT	[382]
	Ag‐SnO_2_	NO_2_	10 ppb–100 ppm	RT	[383]
	Pt‐loaded SnO_2_	H_2_S	10 ppb–10 ppm	RT	[384]
	Au‐loaded WS_2_/SnO_2_	CO	25 ppb–150 ppm	RT	[385]
	Pd decorated GaN	H_2_	42.2 ppb–10 000 ppm	RT	[386]
	Pd‐rGO	NO_2_	1–50 ppm	RT	[387]
	MTiNCs/Pd	formaldehyde	LOD: 4.51 ppb	RT	[388]
	Au‐Pt/WS_2_	NO_2_	ppb‐level	RT	[389]
Photoactivation	Zn‐doped In_2_O_3_	NO_2_	LOD: 10 ppm	RT	[390]
	Nb‐MoS_2_	NO_2_	LOD: 117 ppt	RT	[391]
	TiO_2_/SnO_2_	NO_x_	LOD: 4 ppb (NO)/ 10 ppb (NO_2_)	RT	[361]
	TiO_2_@NGQDs (nitrogen‐doped graphene quantum dots)	NO	10–100 ppm	RT	[392]
	B‐TiO_2_/SnS_2_	acetone	LOD: 757 ppb	RT	[393]
	SnO_2_‐CuO & ZnO‐CuO	H_2_S	25 ppm	RT	[394]
	MAPbI_3_	NH_3_	10 ppm	RT	[395]
	MoS_2_/WSe_2_	NO	0.1–10 ppm	RT	[396]
	ZnO	NO_2_	5–50 ppm	RT	[397]
	rGO@ZnO_1‐x_	NO_2_	ppb‐level	RT	[360]
	In_2_O_3_/CsPbBr_3_	formaldehyde	ppb‐level	RT	[398]
	ZnO/MXene	formaldehyde	100 ppm	RT	[399]
	Cellulose/SnO_2_	NO_2_	100 ppb	RT	[400]
	NiO/CdS	triethylamine	27.8–100 ppm	RT	[401]
	In_2_O_3_@ZnO	NO_2_	Sub‐ppm to 5 ppm	RT	[402]
	TiO_2_/GaN	O_2_	1%–25%	RT	[403]
	PTCDA/ZnO	NO_2_	7.4 ppb	RT	[404]

### 5.3 Development of Chemically and Thermally Stable Integrated Components

The long‐term stability of gas sensors remains a major challenge hindering their commercialization. To improve sensor performance and reliability, optimization of both heaters and contact electrodes is essential. Since most sensors operate at elevated temperatures, the heater and contact electrode materials must exhibit excellent stability. In heater design and fabrication, material selection and structural optimization are critical determinants of thermal performance. An ideal heating element should meet the following requirements: 1) maintain a linear resistance–temperature relationship within the operating temperature range; 2) achieve uniform temperature distribution with low power consumption; 3) preserve stable physicochemical properties throughout its service life.^[^ ^405^ ^]^ These performance characteristics depend on several key material parameters, including but not limited to resistivity, thermal conductivity, thermal expansion coefficient, melting point, Poisson's ratio, and Young's modulus.^[^ ^406^ ^]^

Although numerous materials have been investigated, platinum remains the predominant choice, yet with considerable room for optimization. The primary challenge lies in its poor adhesion to conventional silicon substrates. This can be mitigated by depositing a thin metal sublayer (typically Ti or Ta) between Pt and the substrate. The sublayer not only provides stability and low contact resistance but also retards Pt degradation. However, the sublayer thickness must be carefully controlled, as excessive thickness may accelerate degradation and compromise device reliability.^[^ ^407^ ^]^ Nevertheless, this approach exhibits diminished effectiveness at elevated temperatures, necessitating substrate‐level optimization. The substrate plays a crucial role in Pt adhesion since adhesion results from interfacial interactions between materials. Compared to traditional silicon substrates, ceramic substrates such as alumina offer superior adhesion and thermal stability.^[^ ^408^ ^]^ A.A. Vasiliev et al.^[^ ^409^ ^]^ demonstrated that thin‐film Pt deposited on alumina substrates maintains stable operation at temperatures up to 600 °C for several years after annealing at 850‐1000 °C. To further enhance the high‐temperature stability of Pt films, refractory materials (e.g., Al_2_O_3_, Si_3_N_4_, or TiO_2_) can be deposited as protective barrier layers to shield the Pt from gaseous environments. Alloying Pt with metals such as Rh or Ta can also improve thermal stability by suppressing high‐temperature diffusion and recrystallization.^[^ ^410^ ^]^ Alternative high‐melting‐point metals like Mo and tungsten (W) are also promising candidates. Mo exhibits a high melting point (2623 °C) and low thermal expansion coefficient, while W‐based heaters are particularly suitable for gas sensors operating in extreme environments due to their exceptional temperature resistance.

**Figure 12:**
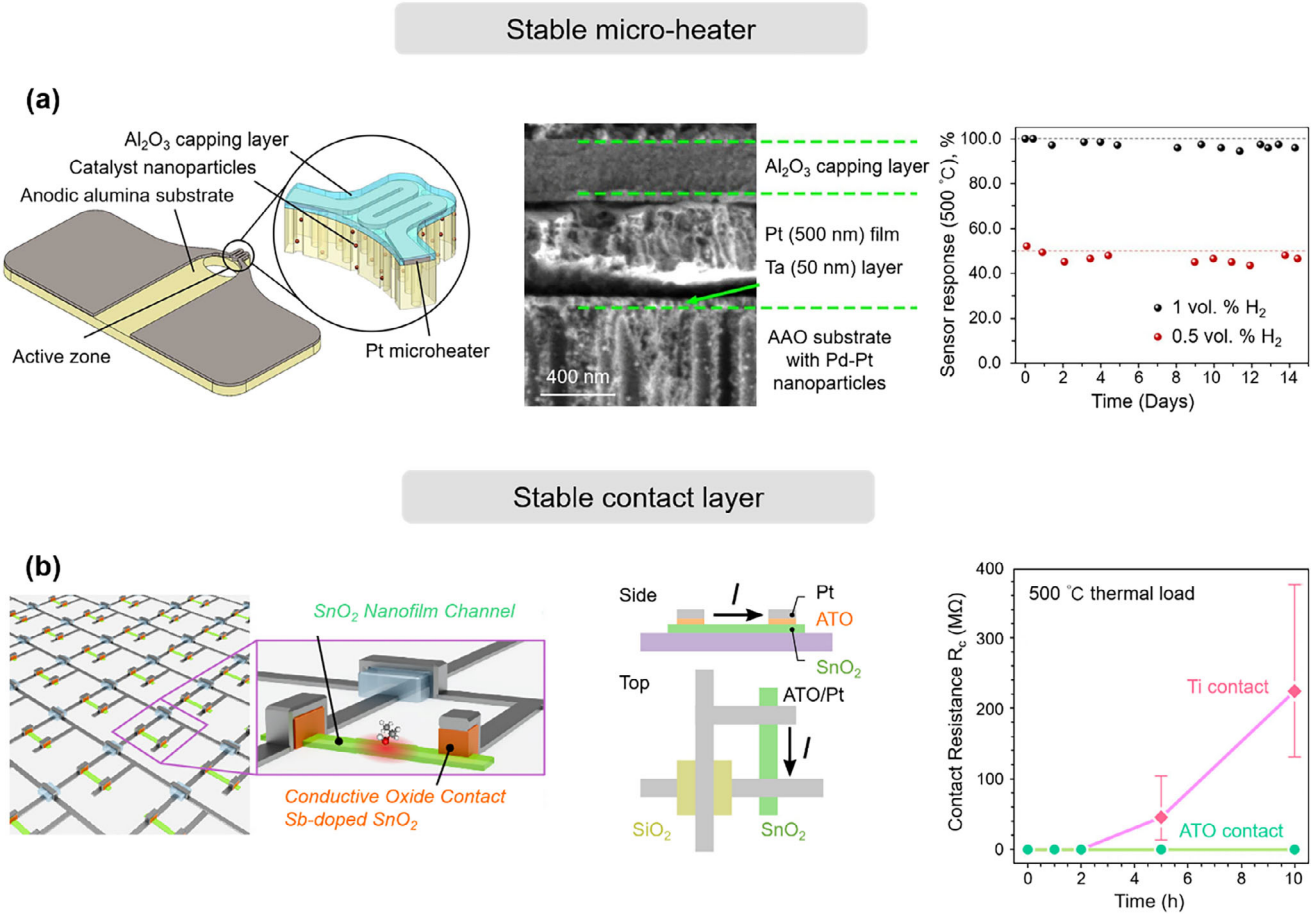
Development of chemically and thermally stable integrated components. a) The structure of the MEMS cantilever gas sensor (left). Fabrication of two‐dimensional MoS_2_ nanosheet‐based microcantilever sensors and their long‐term stability in resonance frequency response curves (right). Reproduced with permission.^[^ ^412^ ^]^ Copyright 2022, American Chemical Society. b) Schematic illustrations of lateral SnO_2_ nanofilm channel sensor devices using ATO as contact electrodes (left). Contact resistance (*R*_c_) of devices with ATO or Ti contacts as a function of thermal load time at 500 °C in ambient air (right). Reproduced with permission.^[^ ^413^ ^]^ Copyright 2022, American Chemical Society.

Conventional single‐layer structures are clearly inadequate for long‐term stability requirements. In contrast, multilayer architectures can significantly improve heater performance by enhancing material adhesion, suppressing oxidation, and retarding degradation.^[^ ^411^ ^]^ As shown in **Figure** **12**a, the optimized multilayer structure employs an anodic aluminum oxide (AAO) substrate for superior thermal stability and mechanical strength.^[^ ^412^ ^]^ A tantalum adhesion layer not only strengthens the metal film's interfacial bonding but also effectively inhibits Pt atomic diffusion. Finally, an alumina protective layer is sputtered onto the Pt surface to isolate environmental interference while further reducing Pt diffusion mobility. This multilayer configuration achieves exceptional performance with minimal resistance drift (1.0 ± 0.3 % per month) and outstanding long‐term stability (more than 1 year at 500 °C).

The stability of contact electrode materials is equally critical for sensor performance. High‐stability contact materials enable low‐resistance ohmic contacts with sensing materials while minimizing contact resistance fluctuations. In oxide semiconductor sensors, traditional metal electrodes (Ti, Al, or Cr) perform well at room temperature but readily oxidize to form insulating or semiconducting phases under high‐temperature operation, leading to significant performance degradation. In contrast, heavily antimony‐doped tin oxide (ATO), a wide‐bandgap oxide semiconductor, demonstrates remarkable high‐temperature stability, maintaining stable conductivity without oxidation at temperatures up to 1000 °C. As shown in Figure 12b, Honda et al.^[^ ^413^ ^]^ compared Ti and ATO electrodes in SnO_2_ sensor arrays, revealing that while Ti electrodes exhibited rapid resistance increase due to oxidation, ATO electrodes maintained stable contact characteristics under prolonged high‐temperature operation.

Beyond material selection, process optimization is crucial for enhancing device stability. Appropriate annealing treatments can effectively relieve internal stresses and improve interfacial properties.^[^ ^414^ ^]^ For instance, Ran et al.^[^ ^415^ ^]^ achieved stable ohmic contacts between Au electrodes and sensing materials through 1‐h annealing at 400 °C, thereby preventing localized heating caused by contact resistance instability.^[^ ^416^ ^]^ Substrate pretreatment is another critical factor, as surface flatness and cleanliness directly affect film adhesion quality and interface characteristics. To minimize environmental impacts, fabricated contact layers should ideally be handled and stored at room temperature.^[^ ^417^ ^]^ From a structural perspective, cantilever‐type heater designs can effectively mitigate mechanical stress concentration during thermal cycling. This configuration accommodates thermal expansion/contraction, significantly reducing deformation and bending while extending device lifetime. By systematically addressing material selection, process optimization, and structural design, the long‐term operational stability of gas sensors can be substantially improved.

### 5.4 Accelerated Aging Treatment Prior to Use

Pre‐aging treatment prior to sensor deployment is of significant importance for obtaining stable response signals and avoiding initial signal drift. Artificially simulating the environmental stress experienced by the sensor during long‐term operation prior to testing, thus achieving accelerated aging, has been proven to be an effective strategy for improving sensor stability. This process can effectively remove surface contaminants and residual impurities from the manufacturing process, while also stabilizing grain structures during the pretreatment phase, thereby improving long‐term sensor stability. Although systematic studies on the effects of accelerated aging on gas sensor performance remain relatively limited, substantial literature has demonstrated that well‐designed aging processes can significantly enhance device stability and repeatability.^[^ ^418^, ^419^, ^420^, ^421^ ^]^

**Figure 13:**
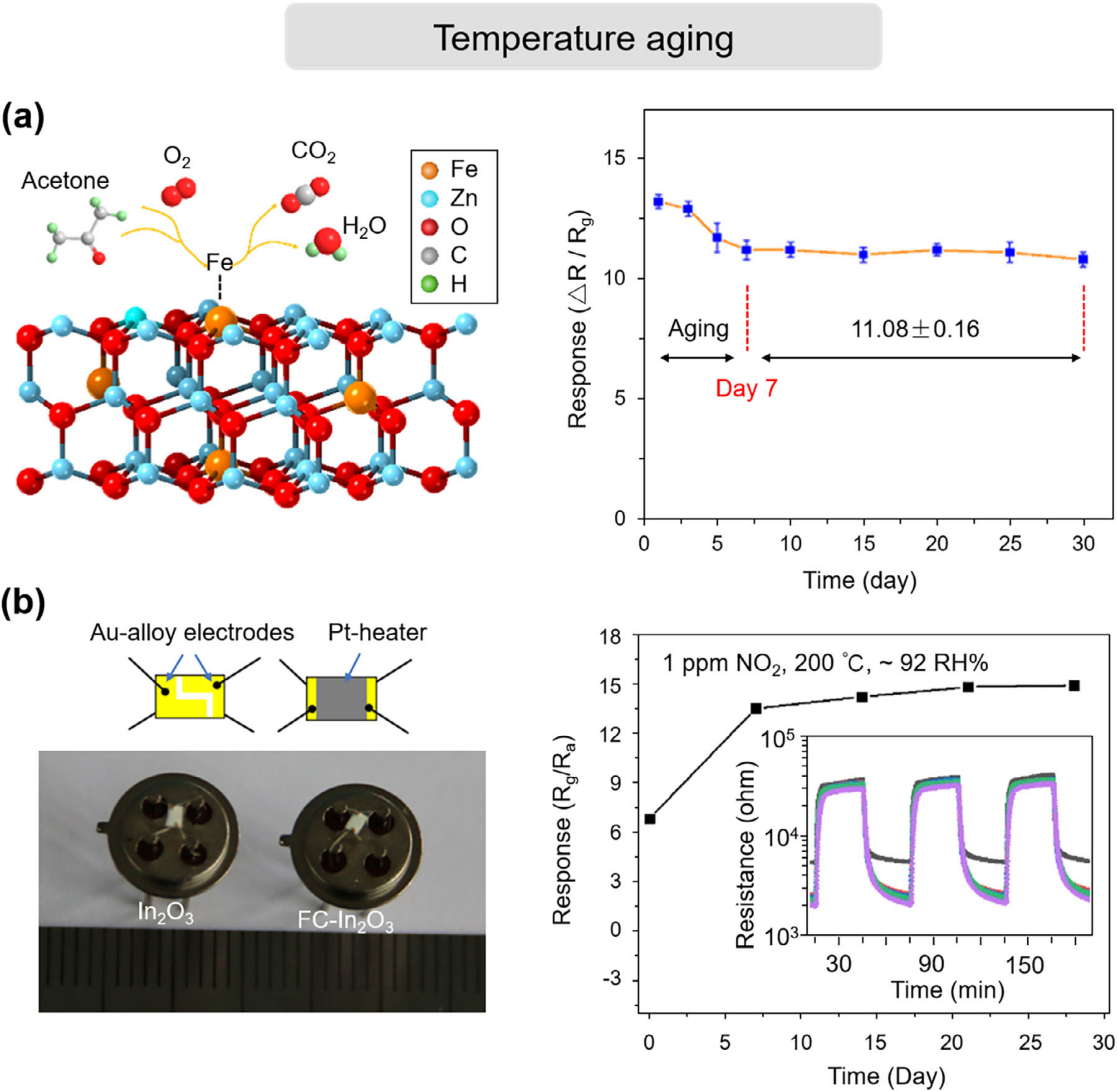
Accelerated aging treatment prior to use. a) Reaction illustration of FZO‐6 for acetone sensing (left). Long‐term stability of FZO‐6 toward 5 ppm acetone vapor at 210 °C and 30% RH (right). Reproduced with permission.^[^ ^422^ ^]^ Copyright 2024, Elsevier. b) Schematic diagram of the electrode and photograph of the fabricated sensors (left). Long‐term stability measurement of CF‐In_2_O_3_ at ∼92% RH in the testing chamber (right). Reproduced with permission.^[^ ^423^ ^]^ Copyright 2021, Elsevier.

For instance, in the work by Shen et al.,^[^ ^422^ ^]^ the sensing performance of their fabricated Fe‐ZnO composite shell MEMS gas sensor gradually declined during the first seven days, while stabilizing with minimal fluctuation during days 7–30 (**Figure** **13**a). The authors attributed the initial performance decrease to the device being in a typical aging phase–the common unstable initial period of semiconductor devices. These results confirm the regular pattern of performance, first decreasing and then stabilizing during the aging process. Du et al.^[^ ^423^ ^]^ observed similar phenomena in long‐term stability tests of In_2_O_3_ sensors. During the 28‐day experimental period, apart from the initial stage, the device's baseline, conductivity response, and recovery characteristics all maintained high consistency, indicating excellent stability (Figure 13b). The authors noted that the instability of newly fabricated devices mainly originates from unstable surface states caused by defects or adsorbed impurities, and as aging progresses, these factors are gradually repaired or passivated, thereby achieving performance stabilization. These studies collectively validate the significant role of pre‐aging in enhancing the long‐term stability of gas sensors.

Pre‐aging is typically conducted in high‐temperature dry environments, though some sensors undergo pre‐aging in high‐humidity conditions, particularly those intended for operation in humid environments.^[^ ^424^ ^]^ This wet aging helps improve the sensor's sensitivity and stability in moist conditions.^[^ ^425^, ^426^ ^]^ Especially for sensing layers containing Pt or Au, exposure to high‐concentration water vapor for several weeks can enable stable operation under certain humidity ranges. During this process, the sensing film forms strong bonds with hydroxyl ions, reducing the number of reversible water absorption sites and thereby rendering the sensor humidity‐resistant. It should be noted that pre‐aging must be conducted at appropriate temperatures and durations; otherwise, it may cause issues such as baseline drift.^[^ ^427^ ^]^ In summary, rationally designing the aging process according to the sensor's actual working environment represents an effective strategy for achieving highly reliable sensing in complex environments.

### 5.5 Introduction of Filters

To enhance the stability of gas sensors, employing various filter materials to remove moisture or interfering gas molecules and regulating the gas composition reaching the sensing materials has proven to be an effective strategy.^[^ ^428^, ^429^, ^430^, ^431^ ^]^ Filter materials are typically positioned at the gas inlet of the sensor or directly coated on the sensing element to optimize the composition and concentration of analytes in mixed gases before they contact the sensing layer, thereby improving the detection environment.^[^ ^432^, ^433^ ^]^

**Figure 14:**
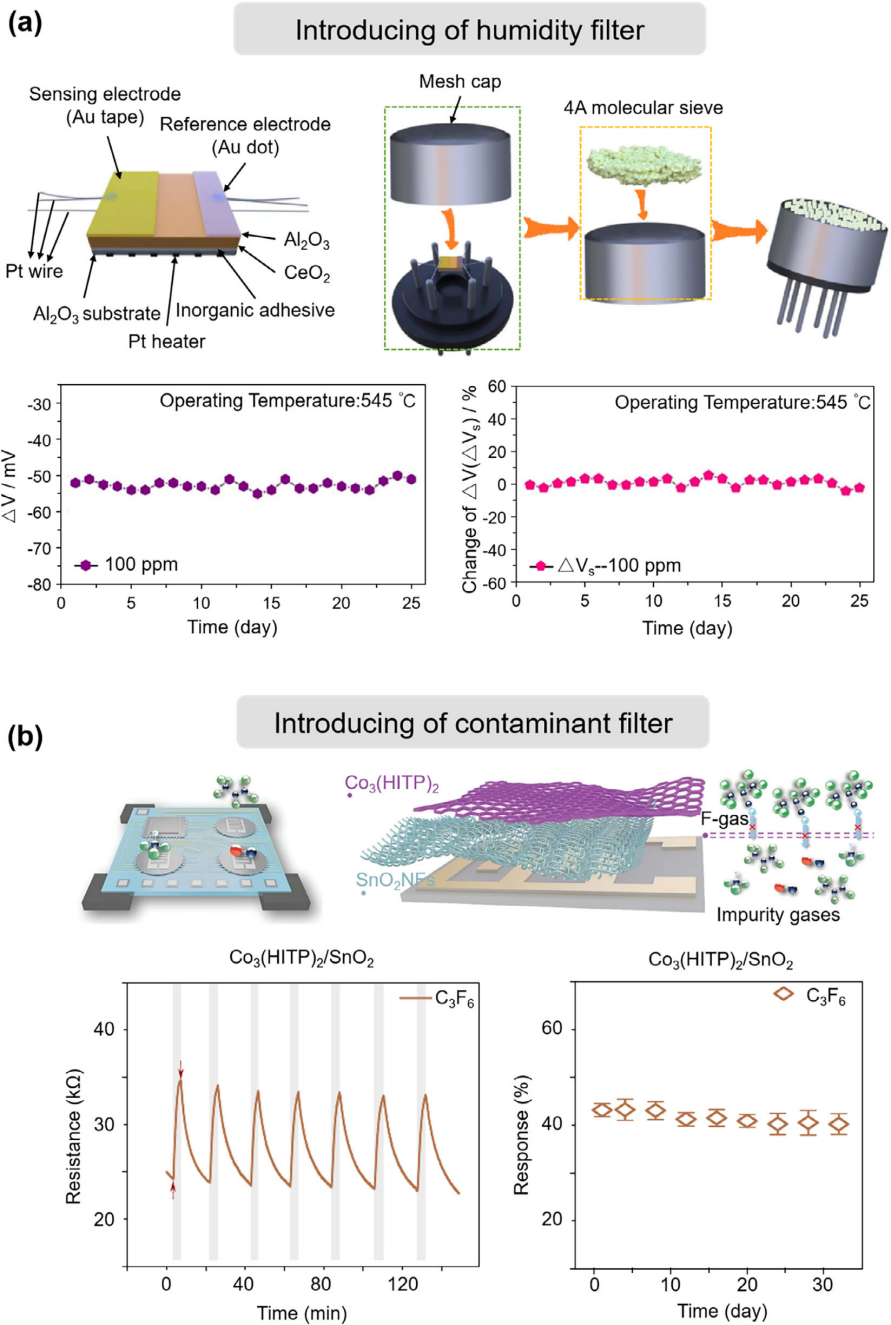
Introduction of filters. a) Structure of the fabricated sensor and schematic illustration of the integration of the mesh coated with a 4A molecular sieve filter layer and the sensor (upper). Response values and rate of change to 100 ppm methanol over a 25‐day test period (lower). Reproduced with permission.^[^ ^437^ ^]^ Copyright 2022, American Chemical Society. b) Schematic illustration of the bilayer sensor array and the Co_3_(HITP)_2_‐coated SnO_2_ bilayer sensor with its filtration effect (upper). Dynamic response curves and long‐term stability of the Co_3_(HITP)_2_/MoS_2_‐SnO_2_ sensor at room temperature under UV irradiation (analyte: 50 ppm C_3_F_6_) (lower). Reproduced with permission.^[^ ^444^ ^]^ Copyright 2024, Wiley‐VCH.

Given that some gas sensors exhibit high sensitivity to humidity, variations in environmental humidity can lead to significant performance fluctuations, severely compromising stability.^[^ ^434^ ^]^ When the sensor itself lacks adequate moisture resistance, removing moisture becomes crucial for accurate target gas detection.^[^ ^435^ ^]^ In this context, filter materials with hygroscopic properties can effectively mitigate moisture interference.^[^ ^436^ ^]^ For instance, Wang et al.^[^ ^437^ ^]^ significantly improved the humidity resistance and long‐term stability of gas sensors by incorporating a zeolite molecular sieve filter layer (**Figure** **14**a). Similarly, Hwang et al.^[^ ^438^ ^]^ developed a microporous polydimethylsiloxane (PDMS) filter that combines moisture resistance with selectivity enhancement, reducing relative humidity by up to 61.05%. However, it should be noted that adsorption‐based filters are prone to gas saturation during operation, affecting their sustained effectiveness. To prolong filter lifespan and maintain adsorption capacity, periodic thermal regeneration using microheaters is often employed to enhance system stability and durability.^[^ ^439^, ^440^ ^]^ Common adsorbents such as zeolites, silica gel, and calcium chloride are susceptible to saturation during long‐term operation and exhibit limited regeneration performance.^[^ ^441^ ^]^

Additionally, some filters can remove pollutant gases and particulate matter, effectively preventing contaminants from reaching the sensor surface and thereby improving selectivity and operational stability.^[^ ^442^ ^]^ For example, particle filters made of polytetrafluoroethylene (PTFE) fibers can efficiently block large‐molecule pollutants, while activated carbon filters remove VOCs from the air, helping maintain baseline signal stability.^[^ ^443^ ^]^ Wu et al.^[^ ^444^ ^]^ proposed a filtration‐sensing strategy based on two‐dimensional MOFs, constructing a bilayer structure consisting of a Co_3_(HITP)_2_ overlayer and a SnO_2_ nanofiber sensing layer (Figure 14b). This approach significantly enhanced room‐temperature detection performance for trace impurity gases under strong fluorinated gas interference. The strategy relies on the MOF's microporous structure for gas diffusion regulation and atomic‐level adsorption interactions, enabling highly selective and sensitive detection of target gases in complex gas mixtures. Similarly, Zeng et al.^[^ ^445^ ^]^ printed HZSM‐5 zeolite films on Pd‐WO_3_ sensing layers to achieve selective CO detection over VOCs. By using Pt/HZSM‐5, the sensor remained selective and sensitive to methanol, even in the presence of high concentrations of CO, as CO was converted into CO_2_. They explored zeolite coatings as a strategy to enhance WO_3_ sensor selectivity.

In summary, as a critical functional component of MEMS gas sensors, filter layers not only play a pivotal role in improving environmental adaptability, enhancing selectivity, and ensuring long‐term stability but also provide new insights for achieving highly reliable detection in diverse and complex atmospheres. Future research should further focus on the design and integration of efficient, multifunctional, and regenerable filter materials to meet the evolving demands for filtration performance.

## 6 Summary

MEMS gas sensors are increasingly central to modern sensing technologies, with applications spanning environmental monitoring, industrial safety, and healthcare. Although their miniaturization and high sensitivity offer significant advantages, maintaining long‐term operational stability, particularly under harsh conditions, remains a critical challenge. This review has systematically outlined the structural design principles, degradation mechanisms, and recent advances aimed at enhancing the long‐term stability of MEMS gas sensors. Chemical degradation, driven by factors such as UV exposure, humidity, and gas‐induced aging, alongside physical damage from film cracking, delamination, and thermal stress on microheaters and electrodes, all contribute to performance deterioration over time. In response, innovative strategies have emerged, focusing on chemically and thermally stable materials, advanced film engineering, and robust structural designs. The integration of single‐crystal materials, 2D nanomaterials, post‐treatment processes, and moisture barriers has notably improved performance stability. Meanwhile, developments in electrode design and low‐temperature operation approaches, such as noble metal catalysts and photoactivation, point toward next‐generation sensor architectures. These multi‐scale approaches represent significant progress toward stable, high‐performance MEMS gas sensors capable of long‐term deployment in complex and dynamic environments. Continued integration of material innovation with structural engineering will be essential for enabling reliable sensor performance in future practical applications.

## 7 Perspective

The development of MEMS gas sensors can be traced back to the mid‐20th century, with catalytic combustion sensors and semiconductor metal oxide sensors serving as their origins. With the maturation of silicon micromachining technology and CMOS‐compatible processes, MEMS sensors gradually achieved the transition from laboratory research to commercialization. In recent years, sensor architectures have evolved from single elements to multi‐sensor arrays, multimodal fusion, and artificial intelligence (AI)‐assisted analysis, with applications spanning smart cities, industrial safety, environmental monitoring, and numerous other fields. Particularly since 2020, the integration of AI chips with MEMS devices has enabled the emergence of intelligent, networked gas detection systems, demonstrating robust real‐time multi‐gas identification capabilities. Against this backdrop, AI technology is progressively becoming a critical enabler for enhancing the long‐term stability of MEMS gas sensors, permeating multiple aspects such as material design, structural optimization, and manufacturing processes.

In terms of material selection, two‐dimensional materials,^[^ ^446^ ^]^ single‐crystal materials,^[^ ^273^ ^]^ and high‐entropy alloys^[^ ^447^ ^]^ are widely regarded as ideal candidates for achieving long‐term reliable operation in MEMS gas sensors due to their excellent chemical and thermal stability. However, the experimental screening process for these material systems is often time‐consuming and resource‐intensive, limiting their efficient development and application. AI algorithms, particularly those in machine learning and high‐throughput computational fields, provide an efficient approach for screening novel materials and predicting their performance. By constructing models based on characteristic parameters such as crystal structure, charge distribution, and reaction energy barriers, AI algorithms can effectively evaluate the chemical and thermodynamic stability of candidate materials under high‐temperature, high‐humidity, or corrosive environments, accelerating the identification of stable materials. Furthermore, AI can assist in optimizing material synthesis conditions, improving experimental efficiency, and reproducibility.

At the device structural design level, nano/micro‐structural optimization^[^ ^448^ ^]^ based on intrinsic material properties (e.g., suspended structure designs) can achieve targeted performance enhancement and improved environmental adaptability. Additionally, the introduction of multilayer structures^[^ ^449^ ^]^ (including adhesion layers, filtration layers, and protective layers) not only enhances structural stability but also provides moisture resistance, dust prevention, and shielding against specific interfering gases, thereby significantly delaying device aging. Particularly noteworthy is the bioinspired design of bionic sensor structures,^[^ ^450^ ^]^ which simulates the highly efficient and stable sensing systems in nature and exhibits unique advantages in signal stability and structural durability, emerging as a promising direction in current sensor structure engineering research. In such structural optimization processes, AI also plays a pivotal role. Through data analysis and modeling, AI algorithms can help identify stress concentration regions and potential failure modes during operation, providing decision‐making support for structural reliability design.

Regarding manufacturing processes, advanced fabrication methods such as photolithography,^[^ ^451^ ^]^ 3D printing,^[^ ^452^ ^]^ and in‐situ growth,^[^ ^453^ ^]^ characterized by precise controllability and potential for mass production, are recommended to ensure the dimensional accuracy of devices and the consistency of performance parameters. Simultaneously, incorporating AI into production processes for parameter optimization and path planning can further improve yield rates and structural uniformity.

**Figure 15:**
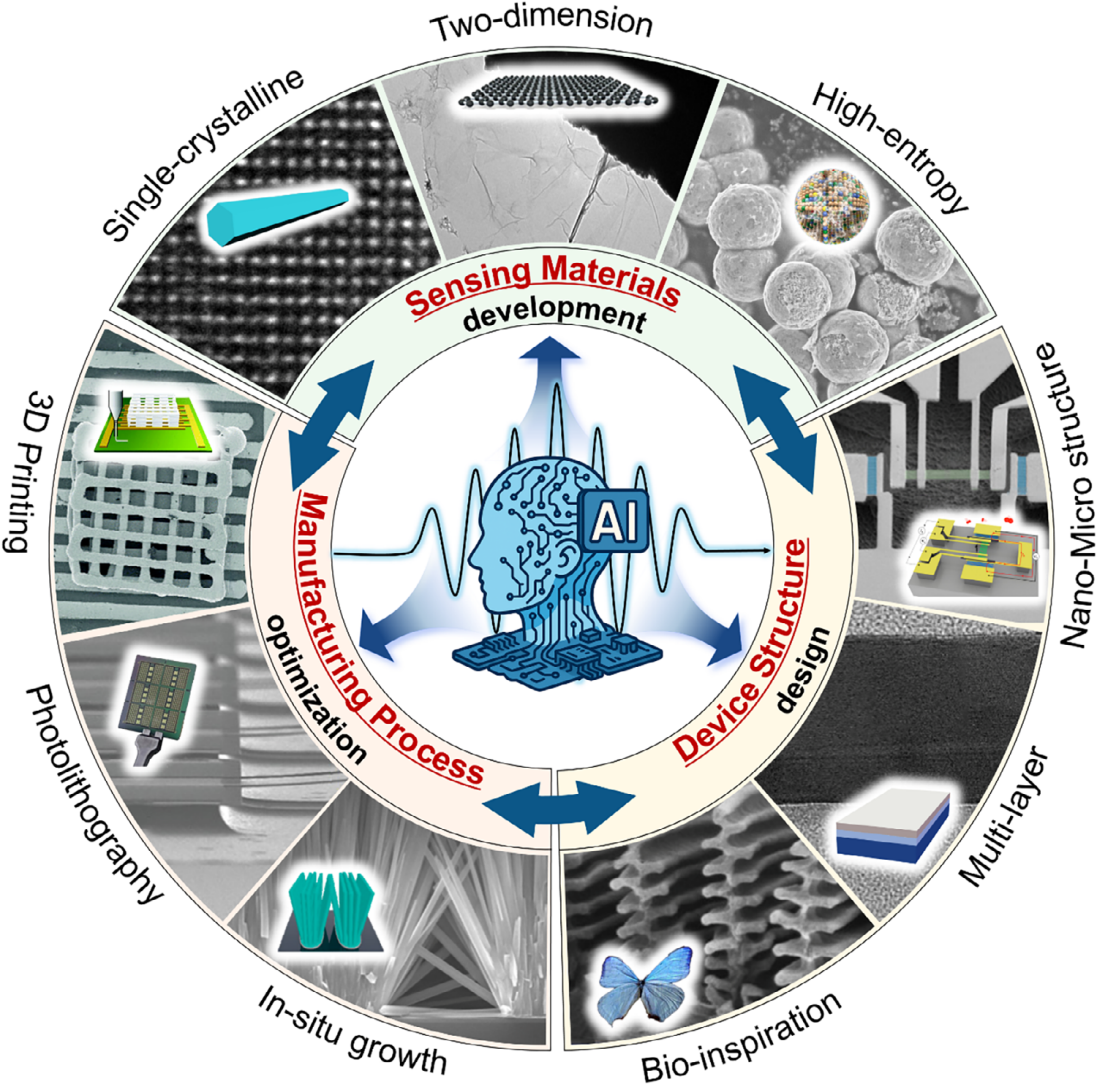
Strategies for enhancing the long‐term stability of MEMS gas sensors.

The synergistic optimization of “materials‐structure‐process,” supplemented by AI‐assisted design and manufacturing (**Figure** **15**), constitutes a key strategy for enhancing the long‐term stability of MEMS gas sensors. However, the performance stability of MEMS gas sensors faces more severe challenges under extreme environmental conditions (e.g., high temperature, high humidity, and strong radiation), where conventional stabilization methods often fail to maintain long‐term effectiveness. Future research directions may focus on developing self‐regulating sensing systems through: 1) exploring materials with self‐healing capabilities or ultrastability to resist long‐term environmental degradation; 2) integrating artificial intelligence algorithms and data correction techniques to enhance sensor anti‐interference capability and long‐term data reliability.

## Conflict of Interest

The authors declare no conflict of interest.
